# Jointly creating digital abstracts: dealing with synonymy and polysemy

**DOI:** 10.1186/1756-0500-5-601

**Published:** 2012-10-30

**Authors:** Steven Vercruysse, Martin Kuiper

**Affiliations:** 1 Systems Biology group, Department of Biology, Norwegian University of Science and Technology, Høgskoleringen 5, 7491 Trondheim, Norway

**Keywords:** Structured digital abstract, Biocuration, Community curation, Ontology, Controlled vocabulary

## Abstract

**Background:**

Ideally each Life Science article should get a ‘structured digital abstract’. This is a structured summary of the paper’s findings that is both human-verified and machine-readable. But articles can contain a large variety of information types and contextual details that all need to be reconciled with appropriate names, terms and identifiers, which poses a challenge to any curator. Current approaches mostly use tagging or limited entry-forms for semantic encoding.

**Findings:**

We implemented a ‘controlled language’ as a more expressive representation method. We studied how usable this format was for wet-lab-biologists that volunteered as curators. We assessed some issues that arise with the usability of ontologies and other controlled vocabularies, for the encoding of structured information by ‘untrained’ curators. We take a user-oriented viewpoint, and make recommendations that may prove useful for creating a better curation environment: one that can engage a large community of volunteer curators.

**Conclusions:**

Entering information in a biocuration environment could improve in expressiveness and user-friendliness, if curators would be enabled to use synonymous and polysemous terms literally, whereby each term stays linked to an identifier.

## Findings

### Introduction

The Life Sciences are producing vast amounts of information. Each year, over half a million new publications is indexed by PubMed/Medline, and this volume keeps growing increasingly faster. All this information can only be processed effectively with computer assistance. However, most knowledge is only reported through natural language, a format that remains fairly opaque to computers despite flourishing text-mining research [1,2]. Comprehensive and accurate digital formalisation of the published information still needs human intervention, a process called *manual curation*. But it was shown that curators working in small, focused groups (like institutes) don’t have the capacity to keep up with the enormous growth of new findings [3]. This calls for a *crowdsourced* setup: a large, distributed community of scientists that collectively curates on a part-time, volunteer basis [4-7].

As has been argued, every publication should best become accompanied by a manually created, or at least validated, *structured digital abstract* (SDA) [7-9]. A few years ago the journal FEBS Letters launched an initiative to let authors create digital abstracts when they submitted a paper [7]. An Excel-sheet was provided with a number of mandatory and optional columns as a template, and the focus was mostly on curating protein interactions. In fact, many researchers in diverse life science areas are performing a related task as part of their daily work. While reading a scientific paper they often take concise notes, which summarize the paper’s main findings for later reference. Although informal, these notes actually cover a much larger variety of topics, and contain more flexible levels of detail than spreadsheets or other pre-designed forms. It would constitute a considerable potential community resource if there would be mechanisms allowing scientists to store ‘full’ digital abstracts, i.e. summaries that cover a wide range of topics and detail, in a semantically sound format. Moreover, this formalisation would allow scientists to share notes/summaries, improve each others’ summaries, and query them. This would enable the crowdsourced creation of comprehensive digital abstracts.

As a first step beyond the spreadsheet, one may use a *controlled language* as a means of semantic encoding. Controlled languages define a restricted subset of English and are used for semantification in several fields, e.g. Attempto Controlled English (ACE) [10,11], Common Logic Controlled English (CLCE) [12], Biological Expression Language (BEL) [13]. Each one consists of: 1) a *controlled vocabulary*, which is its set of unique terms that each represent a specific concept in a domain; and 2) a *controlled syntax*, defining how users may combine the terms to construct the formal, *controlled sentences* of the language, that are recognised by a specific parser algorithm. A controlled language is a magnitude more flexible than predesigned forms, as a controlled sentence can cover any topic, and it supports the addition of sub-structures.

In order to develop an overview of the hurdles towards crowdsourced full digital abstracts, we wanted to gain insights in how life-scientists would interact with a custom-made controlled language. We created a controlled language by using terminology from a selection of ontologies and gene lists, and by defining a syntax to support a variety of sentence types. We embedded it in a local web-application, provided a visualizer with some similarities to the recently published OLSVis [14,15], organised test-sessions with volunteers, and studied user feedback. In this paper we first briefly describe this setup and how it was received. Then we report on what we experienced in this pilot study, and come with specific recommendations that can be useful to create an intuitive curation environment that attracts a larger crowd of volunteer curators.

### Controlled-language and test-application

We designed a *controlled language* for capturing a variety of biological information types formally. Firstly, to populate its *controlled vocabulary* (CV) we chose a number of ontologies and gene lists of interest to our test-users: GO, Plant Ontology, PATO, Arabidopsis genes, human genes, etc. Spaces in multiword terms were replaced by “_” for correct automated parsing. To represent a concept (gene, tissue, etc.) that is described in a paper, a curator must find the intended canonical term (database entry) in a source vocabulary. This *normalisation* is the first important step in the curation process, and is more elaborately discussed e.g. in [16,17], from the perspective of text-mining, which can also assist curation. For our project, biologist curators used existing online resources where they found the terms’ definition, synonyms, and other relevant metadata to help disambiguation. Our software also provided an autocomplete feature that helped with entering these terms correctly in the input text-field. By using a CV, users are limited to a set of standardized terms, which enables interoperability. However, during the curation process curators typically discover concepts that are not yet covered by terms in these resources. But the integration of new terms into ontologies requires a lengthy process of term submission, committee review and approval. Therefore, in order not to impede the curation process we chose to enable users to add and immediately use these extra terms in the curation system’s internal, merged CV.

Secondly, to connect the terms through a *controlled syntax* we defined a comprehensive set of syntactic patterns, which describe the types of allowed controlled-sentences, i.e. how terms can be put together in combination with fixed keywords. In our case, the keywords are mainly symbols that express some relation, resulting in a language that has some similarities with BEL [13]. In brief, supported information types include protein interactions, transgenic modifications, phenotype effects, environmental cues, uncertain facts, and many more. As an example, the line “Abc[P] **-**> Xyz @L nucleus @T G1.end” would read as: phosphorylated Abc stimulates Xyz, at location nucleus, and at time end of G1-phase.

**Figure 1:**
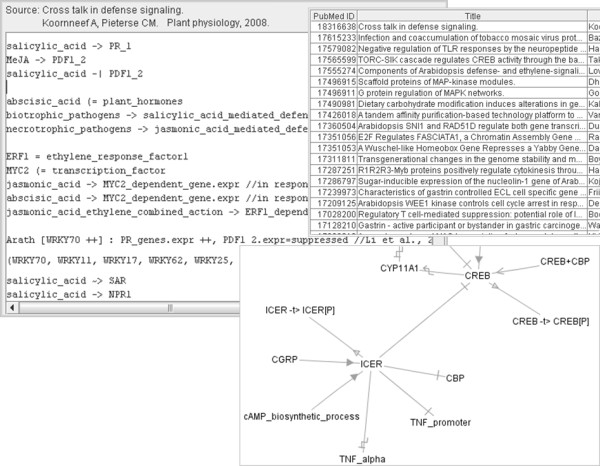
**Screenshots of the ‘MineMap’ test-application.** Clockwise: input box for one article’s digital abstract (cooperatively created); partial list of curated papers; part of a visualised network.

We implemented a parser for this language and created a local web-application/Java-applet *MineMap,* see also Figure 1. By entering a PubMed-identifier a new digital abstract is started, or an existing one is shown. The digital abstract is shown as an editable text field where each line can contain a controlled sentence. Users can inspect and edit each other’s work, and changes are logged and linked to the logged-in user. When a user types, an autocomplete-panel assists with vocabulary lookup, including synonyms, and after pressing Enter the preferred, official term is placed in the sentence. Information is stored, via PHP, in a MySQL database that has no problem with conflicting information. In order to attract test-users, we also implemented a module that visualises all curated entities and relationships as a graph of related terms (as in [14,15]).

Our volunteer-curators were mainly wet-lab biologists that wished to encode information on a variety of topics: the Arabidopsis cell cycle; leaf development in both wild-type and transgenic plants; effects of viral infection in both Arabidopsis and Tobacco; or signaling triggered by the human stomach hormone gastrin. For most biologists, this was their first curation experience. We organised two jamboree sessions and also guided individual curators. We collected feedback from over two dozen users, detailing their experiences with the system. The curators extracted information from the abstracts and full-text bodies of 56 papers. This amounted to 1367 curated statements (this includes a few comments and some declarations), see also Additional file 1.

The curators in general appreciated the concept of community-based digital-abstract creation, especially since the results of their efforts could also immediately be visualised as a graph with terms linked through relationships. The curators also liked the fact that the method enabled to combine several information types into a single digital-abstract per article. But we also observed concerns: additional improvements were clearly needed in order for a controlled-language-based input to become user-friendly enough to meet a larger community of ‘lay-men’ curators. Based on this experience, we come to a number of recommendations for making such a system more user-amenable, focusing on a number of suggestions for *controlled*-*vocabulary*-related improvements that can make the creation of digital abstracts more user-friendly.

### Insights: solving synonymy and polysemy ‘behind the screen’ to please biocurators

Note: *Synonymy* is the situation where one concept (a meaning/idea) is represented by several terms: synonyms, e.g. in Table 1: ‘CDC2’, ‘CDK2’, and ‘CDKA;1’ all refer to the same gene. *Polysemy* is the situation where one term represents several concepts, i.e. a polysemous term has several meanings; e.g. ‘LMR1’ refers to three different genes. Note that the general definition of ‘polysemy’ is used here: the different meanings of a term may be related or unrelated (as in strict polysemy vs. homonymy).

**Table 1:** Illustration of the Life Sciences’ many synonymous and polysemous terms

**Official term or gene symbol**	**Synonyms**	**Ontology or term list**
AT3G48750	CDC2, CDC2A, CDC2AAT, **CDK2**, CDKA1, CDKA;1, Cell Division Control 2, …	A. thaliana
AT1G52340	ABA Deficient 2, ATABA2, ATSDR1, GIN1, Glucose Insensitive 1, Impaired Sucrose Induction 4, ISI4, Salt Resistant 1, SDR1, SIS4, SRE1, Sugar-Insensitive 4, …	A. thaliana
**CDK2**	Cyclin-Dependent Kinase 2	H. sapiens
ABCC8	**MRP8**, HI, PHHI, SUR1, ABC36, HHF1, …	H. sapiens
S100A8	**MRP8**, P8, 60B8AG, CGLA, CAGA, CFAG	H. sapiens
AATK	AATYK, KIAA0641, LMTK1, **LMR1**, …	H. sapiens
**LMR1**	Leishmaniasis Resistance 1	M. musculus
**LMR1**	CHLREDRAFT_184328	C. reinhardtii
Fruit	achene, berry, capsule, caryopsis, circumcissile capsule, cypsela, drupe, follicle, grain, nut, pod, poricidal capsule, silicula, siliqua, silique, …	Plant Ontology
nodal root	crown root, seminal root	Plant Ontology
erythrocyte	red blood cell, RBC	Cell Type

Many gene aliases and synonyms as used in literature. Terms in bold highlight polysemy conflicts, esp. some terms that are official gene symbols in two species. Note that ‘silique’ is a term used in Arabidopsis research to refer to the plant’s dry seed pods, although the official PO term is ‘fruit’. For *A*. *thaliana* we queried TAIR [18], for *H*. *sapiens*: HUGO [19], for *M*. *musculus* (mouse) and *C*. *reinhardtii*: NCBI [20], for Plant Ontology: PO [21], for Cell Type: OLS [22,23].

#### Creating a clearer curation experience

A controlled language’s vocabulary consists of a list of unique terms that each represents a single meaning. This one-to-one mapping between terms and concepts is essential to make sentences unambiguous. But in the Life Sciences a concept is often represented by several synonyms. Therefore, an authoritative organisation then selects which term is the preferred one and includes it in the official term list (e.g. TAIR, HUGO, PO [18,19,21,24,25]). However, many authors of publications keep using non-preferred terms, for example because it still is the preferred terminology in their own scientific niche or laboratory environment. As a result, life science literature actively uses a vast variety of synonyms [26], see also Table 1. So during literature curation, these synonyms are only used as guides to locate their official term, which is then placed instead of the synonym in the controlled sentence; cf. MineMap’s autosuggestion feature.

However, when official terms replace terminology that was used in the original publication, the link between the digital abstract and its source paper becomes confusing. In test sessions with MineMap, we observed that many biologists started a digital abstract by declaring a list of aliases and abbreviations they were going to use: new terms linked to existing concepts. When composing a digital abstract, users found it a burden to use several official terms that were either awkwardly long or did not correspond to the terms being used by the author in the paper. These official terms may also pose an extra mental workload for curators who want to review digital abstracts created by fellow curators. We believe that this burden is completely unnecessary, and that synonymous terms may very well be used literally (=as they are) in controlled sentences, as long as the terms are disambiguated by their link to a unique semantic identifier (ontology-ID, URI, etc). The solution is to develop a software interface that provides more than just a text-input field for controlled-sentences. It should be designed as an input system that works on two levels. Behind the screens, the controlled language should work on curated statements that are based on semantic identifiers. Meanwhile, the user-interface should show and manage the entered terms that are linked to these identifiers. If dedicated curation software supports such ‘active’ use of a paper’s terminology while curating, digital abstracts can reflect their originating paper more transparently. This can result in a more attractive and clearer curation process, easier cross-checking, higher curation efficiency, and a lower threshold for participation in curation by wet-lab biologists.

#### Enabling dictionary combination

**Figure 2:**
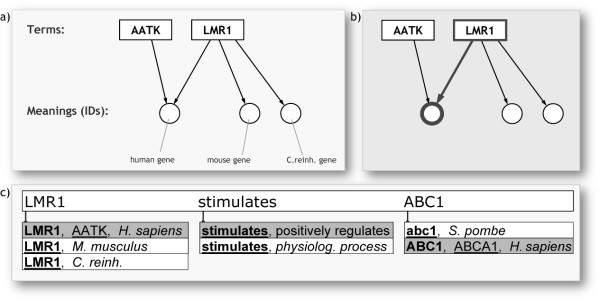
**Terms as guides to meanings, and proposed upgrade for ‘controlled language’ input.** (**a**) Example with two synonyms, of which one is polysemous as well. (**b**-**c**) Although the term AATK is preferred for some human gene, a user can make the link between the non-preferred synonym LMR1 and the same concept. Via an autosuggestion-panel (here a mock-up), the curator would be able to appoint the intended meaning for a term. Underlined terms are official, non-italic terms are synonyms. In italic is extra info for disambiguating polysemous terms.

The life sciences are producing a broad field of knowledge where many concepts from various subfields can in some way be connected with each other. An integrative, more united platform for creating digital abstracts should therefore build on a large, cross-topic vocabulary. This requires support for using terms from many ontologies (see NCBO BioPortal [27]) and gene lists from many different species. Hereby it is unavoidable that term collisions will occur: some terms become polysemous, see e.g. Table 1. Thus also polysemous terms must be supported in curated sentences, as long as they are linked to a specific semantic identifier. The curation platform’s autosuggestion feature can readily be extended to accommodate this, as in Figure 2c. Synonymy, polysemy, and a simple mechanism to deal with this in digital-abstract curation are illustrated in Figure 2.

#### Unification of lexical-class variants

In our system we used a series of symbols to represent relationships, e.g. “->” for “activates”. These were the fixed ‘keywords’ of the controlled language. But this limits the system’s scalability. In the long run there will be more biological relations than symbols that curators can manage, so one should eventually use actual terms to represent the relations. Then a sentence like “A @L B” becomes “A is_located_in B”. But then also, “A -> B @L C” would become “A activates B in C”. Note the use of “in C” here, instead of “is_located_in C”, which points out a duality between a verb-form and a preposition-form for the same concept. In addition, a same argument can be made for the noun/verb case, e.g. for activation/activates in “D enhances the activation of B by A”. We believe that in general, allowing the use of prepositions etc. where one would also use them in a natural language sentence, could make curated sentences easier to create and to re-read. This implies that a curation system could also support synonymy over lexical variants. Also, for many prepositions one would then need polysemy disambiguation support.

We observed that curation with controlled-sentences becomes more difficult the more a sentence looks artificial. So this type of curation can become user-friendlier by a design that allows more natural-language-like aspects, as long as all terms are still disambiguated via links to semantic IDs.

### Summary

**Figure 3:**
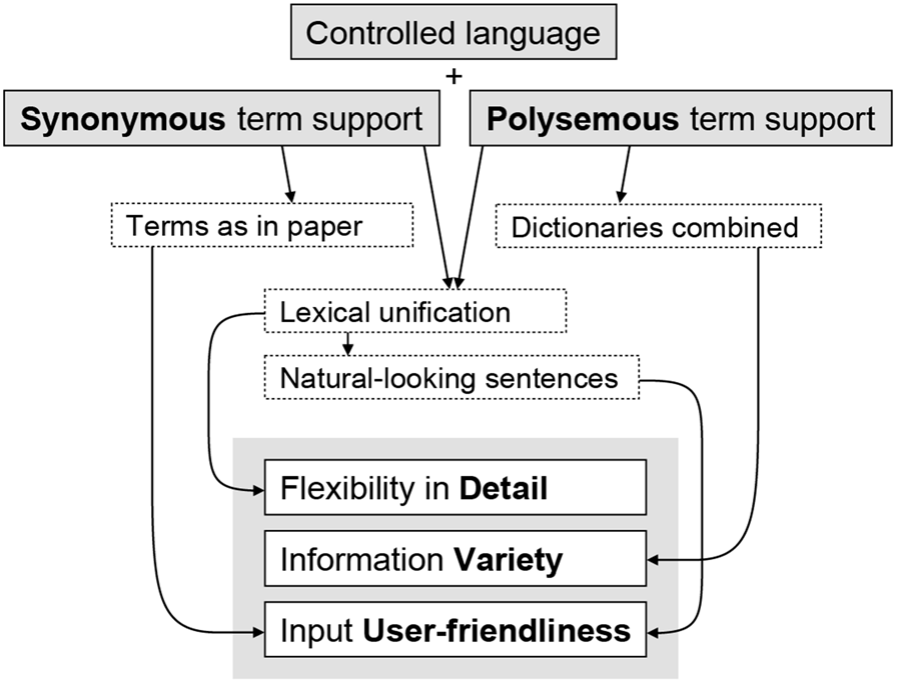
**Benefits of biocuration with support for using synonymous and polysemous terms as they are.** Provided a mechanism for linking each term to a unique identifier, the controlled language could have improved expressiveness and usability.

The life sciences need an information capturing method that can support the creation of digital abstracts with a large variety in content, intuitive and attractive to a larger crowd of curators. A controlled language offers a higher flexibility than forms or spreadsheets, and we implemented one in a test-application. Based on curator experiences, we argued that both expressiveness and user-friendliness could be improved by adding support for the literal but ID-linked use of synonymous and polysemous terms, see Figure 3.

### Implications

In order to process such digital abstracts, an extension must be made to the conventional ‘parsing algorithm’. It should not only parse literal text; it also has to manage the identifier behind each entered term/token. An application can still offer a view-mode that shows official terms instead of those used during curation, because terms are still linked to their official term via a semantic ID. This would be useful for viewing several digital abstracts simultaneously, or for any form of integrative view. Various extra features could further improve the system. As one example: text-mining could identify terms in an article and rank these highest in the autosuggestion panel in Figure 2c.

Note: if at some point in the future, SDA creation would scale up so much that each publication gets an SDA, then still text-mining will be useful. As shown in [17], a hybrid approach of text-mining and manual curation gives more precise results. Also, manual curation can be facilitated by text-mining. Meanwhile, SDAs’ curated facts can be used as training data to improve the text-mining software.

### Future work

After studying vocabulary-related improvements in this short paper, we are currently improving controlled-syntax aspects, likewise aimed at making digital abstract creation more flexible and user-oriented. These improvements will ultimately be made available through a novel web-application.

## Abbreviations

ACE: Attempto Controlled English; BEL: Biological Expression Language; CLCE: Common Logic Controlled English; CV: Controlled vocabulary; HUGO: Human Genome Organisation; GO: Gene Ontology; ID: Identifier; PATO: Phenotype Attribute and Trait Ontology; PO: Plant Ontology; SDA: Structured digital abstract; TAIR: The Arabidopsis Information Resource; URI: Uniform Resource Identifier.

## Competing interests

The authors declare that they have no competing interests.

## Authors’ contributions

SV implemented the MineMap application. MK participated in organising the project. SV and MK drafted the manuscript. Both authors read and approved the final manuscript.

## Supplementary Material

Additional file 1 **Curated data and controlled language specification.** Section A shows all curated statements, grouped per publication identified by PubMed-ID. The statements follow the controlled syntax that is described in Section B. Click here for file
