# Supplementary material for: Jointly creating digital abstracts: dealing with synonymy and polysemy
Source: BMC Res Notes. 2012 Oct 30;5:601. doi: 10.1186/1756-0500-5-601 (PMC3532140; doi:10.1186/1756-0500-5-601)
Supplement: Additional file 1 — Curated data and controlled language specification. Section A shows all curated statements, grouped per publication identified by PubMed-ID. The statements follow the controlled syntax that is described in Section B. [file 1756-0500-5-601-S1.doc]

Supplementary data:

**A) The curation results:**

| **Pubmedid** | **Title** | **authors** | **source** | **Annotationtext** |
| --- | --- | --- | --- | --- |
| 15377755 | The plant-specific cyclin-dependent kinase CDKB1;1 and transcription factor E2Fa-DPa control the balance of mitotically dividing and endoreduplicating cells in Arabidopsis. | Boudolf V, Vlieghe K, Beemster GT, Magyar Z, Torres Acosta JA, Maes S, Van Der Schueren E, Inze D, De Veylder L. | Plant Cell. 2004 Oct;16(10):2683-92. Epub 2004 Sep 17. | SPECIES: Arath //Columbia  SUBJECT: core cell cycle genes  //leaf //PO  //leaf[one_two] //to be taken into consideration by "merging" PO and minemap  //cotyledon //PO  //area //PATO  //cotyledon_abaxial_epidermis //PATO  //leaf_abaxial_epidermis //PATO  //stomatal_index //to include in some phenotype ontology  leaf[one_two]=)leaf  CDKB1;1{AT3G54180}=CYCLIN-DEPENDENT_KINASE_B1;1  Arath[CDKB1;1--]:cotyledon.area=decreased  Arath[CDKB1;1--]:cotyledon_abaxial_epidermis.area=increased // problem: cells are described but tissue are specified  Arath[CDKB1;1--]:cotyledon_abaxial_epidermis.number=decreased  Arath[CDKB1;1--]:cotyledon.stomatal_index=decreased  Arath[CDKB1;1--]:leaf[one_two].area=decreased  Arath[CDKB1;1--]:leaf[one_two].leaf_abaxial_epidermis.area=increased  Arath[CDKB1;1--]:leaf[one_two].leaf_abaxial_epidermis.number=decreased  Arath[CDKB1;1--]:leaf[one_two].stomatal_index=decreased  Arath[CDKB1;1--]:cotyledon.stomatal_complex.structure=abnormal //up to 58.5%, unicellular round or kidney-shaped, no pore  //name:CDKB1;1.N161[lof;D-;transgene;35S]  Arath[CDKB1;1++]:cotyledon.area=equal  Arath[CDKB1;1++]:cotyledon_abaxial_epidermis.area=equal  Arath[CDKB1;1++]:cotyledon_abaxial_epidermis.number=equal,cotyledon.stomatal_index=equal  Arath[CDKB1;1++]:leaf[one_two].leaf.area=equal  Arath[CDKB1;1++]:leaf[one_two].leaf_abaxial_epidermis.area=equal  Arath[CDKB1;1++]:leaf[one_two].leaf_abaxial_epidermis.number=equal  Arath[CDKB1;1++]:leaf[one_two].stomatal_index=equal  //[gof;D+;transgene;35S]  Arath[CDKB1;1--][CDKB1;1++]:cotyledon.stomatal_index=equal  Arath[CDKB1;1--][CDKB1;1++]:cotyledon.stomatal_complex.number=equal  //The DN mutation phenotype is suppressed by overexpressing the wt gene.  Arath[CDKB1;1--][CDKA;1++]:cotyledon.stomatal_index=decreased  Arath[CDKB1;1--][CDKA;1++]:cotyledon.stomatal_complex.number=decreased  //[CDKA;1++] is [gof;D+;transgene;35S]  //The DN mutation phenotype is not suppressed by overexpressing CDKA;1. |
| 16943276 | The HVE/CAND1 gene is required for the early patterning of leaf venation in Arabidopsis. | Alonso-Peral MM, Candela H, del Pozo JC, Martinez-Laborda A, Ponce MR, Micol JL. | Development. 2006 Oct;133(19):3755-66. Epub 2006 Aug 30. | SPECIES: Arath  SUBJECT: Leaf_Development  CAND1{AT2G02560}=cullin_associated_and_neddylation_dissociated  HVE{AT2G02560}=HEMIVENATA  CUL1{AT4G02570}=CULLIN1  AXR6{AT4G02570}=AUXINRESISTANT6  TRN1{AT5G55540}=TORNADO  LOP1=LOPPED1  TRN1=LOP1  CAND1=HVE  CUL1=AXR6  Arath[HVE]<->Arath[CUL1]  //HVE-- is a T-DNA allele  vascular_system (= cell_type  leaf =) cell_type  leaf[one_two] (= leaf  cotyledon (= leaf  cauline_leaf (= leaf  petal (= leaf  sepal (= leaf  vascular_system =) areole  vascular_system =) secondary_vein  vascular_system =) tertiary_vein  vascular_system =) quaternairy_vein  vascular_system =) midvein  mesophyll (= cell_type  spongy_mesophyll (= mesophyll  vascular_system (= cauline_leaf  cauline_leaf_vascular_system (= vascular_system  petal_vascular_system (= vascular_system  sepal_vascular_system (= vascular_system  Arath[HVE--]:leaf[one_two].leaf_area=decreased  Arath[HVE--]:cotyledon.areole_number=2  Arath[HVE--]:cotyledon.vascular_system=open  Arath[HVE--]:leaf[one_two].secondary_vein_number=decreased  Arath[HVE--]:leaf[one_two].tertiary_vein_number=decreased  Arath[HVE--]:leaf[one_two].quaternairy_vein=absent  Arath[HVE--]:cauline_leaf.leaf_area=decreased  Arath[HVE--]:cauline_leaf.vascular_system=equal  Arath[HVE--]:petal_vascular_system=decreased  Arath[HVE--]:sepal_vascular_system=decreased  Arath[HVE--]:leaf[one_two].midvein.vein_thickness=decreased  Arath[HVE--]:leaf[one_two].spongy_mesophyll.air_space=increased  Arath[HVE--][proATHB8_GUS++]:transcription = abnormal  //++ is inserted to be able to implement a GUS-fusion which is not a ++ or a --  //things should be placed more hierachical to make the visualisation less dirty  Arath[HVE--]:leaf.cell_type.mesophyll.spongy_mesophyll.air_space=increased  proHVE=)HVE  Arath[proHVE_GUS++]:transcription = decreased @ leaf @ development  Arath[proHVE_GUS++]:transcription = increased @ vascular_system @ development  Arath[proHVE_GUS++]:transcription = present @ vascular_system @ before_differentiation  // ckeck the way to implement an expression pattern |
| 16786292 | Characterizations of a hypomorphic argonaute1 mutant reveal novel AGO1 functions in Arabidopsis lateral organ development. | Yang L, Huang W, Wang H, Cai R, Xu Y, Huang H. | Plant Mol Biol. 2006 May;61(1-2):63-78. | SPECIES: Arath  SUBJECT: leaf  AGO1{At1g48410}=Argonaute1_RNA_Slicer  AS2{AT1G65620}=asymmetric_leaves2  FIL{AT2G45190}=YAB1_YABBY_gene_family_member  YAB3{AT4G00180}=YABBY_gene_family_member  Arath[AGO1--]:leaf.number--//2-3 rosette leaves before flowering in ago1-37, _  leaf_abaxial_epidermis.leaf_vascular_system.midvein=abnormal//small outgrowths were associated with the midvein _  on the abaxial side of the rosette leaves in ago1-37, whole_plant.size=small//in ago1-8, _  leaf[one_two].shape=needle//in ago1-8, -  cauline_leaf.number--//in ago1-37 secondary inflorescence branches are not always associated with cauline leaves, _  cauline_leaf.shape--//in ago1-37 secondary inflorescence branches are sometimes associated with filamentous organs and sometimes with filamentous organs, _  leaf.shape=narrow//in ago1-37, leaf_adaxial_epidermis.trichomes=present//in ago1-37, leaf_abaxial_epidermis.trichomes=present//in ago1-37 _  leaf.petiole=abnormal  Arath[AS2--]:leaf_epidermis.cell++ @leaf_margin //long straight cell files increased markedly along the sinus of as2-101 leaves  Arath[AGO1--][AS2--]:leaf[one_two].shape=lobate//lobes appear in the first pair of leaves of ago1-37 as2-101 and ago1-27 as2-101, _  leaf.margin=thickened//in ago1-37 as2-101 because of dramatic increase in long straight cell files, _  leaf.polarity=abnormal  Arath[AGO1--][fil--][yab3--]:whole_plant.size=small//ago1-37 fil yab3-2, leaf.shape=needle, cauline_leaf.shape=needle |
| 17351053 | A Wuschel-like Homeobox Gene Represses a Yabby Gene Expression Required for Rice Leaf Development. | Dai M, Hu Y, Zhao Y, Liu H, Zhou DX. | Plant Physiol. 2007 Mar 9;. | SPECIES: Oryza  SUBJECT: Leaf_Development  YAB3 = Oryza.YABBY  WOX3 = Oryza.Wuschel_like_Homeobox  Arath.FIL{AtxGxxxxx} = Arath.Filamentous_flower  Zeama.NS1_2 = Zeama.narrow_sheath_1_2  PRS = Arath.PRESSED_FLOWER  YAB3 =h Zeama.YAB14  YAB3 =h Arath.FIL  WOX3 =h Zeama.NS1_2  KNOX = KNOTTED_LIKE_HOMEOBOX  OSH1  OSH3  KNOX =) (OSH1, OSH3)  YAB3.expr = 0 @ shoot_apical_meristem //in situ hybridisation  WOX3.expr = 0 @ shoot_apical_meristem //in situ hybridisation  YAB3.expr = high @ (leaf_primordial, young_leaves, reproductive_organs)  WOX3.expr = high @ (leaf_primordial, young_leaves, reproductive_organs)  Oryza[YAB3--]: leaf_lamina = twisted, leaf_margin = knotted, ligule = absent, auricle = absent//RNAi; Zhonghua 11 cultivar;  Oryza[YAB3--]: OSH1.expr = induced, OSH1.expr = ectopic  Oryza[YAB3--]: OSH3.expr = induced, OSH3.expr = ectopic  //YAB3 expressed in nucleus  //WOX3 expressed in nucleus  Oryza[YAB3++]: whole_plant = equal //no effect  Oryza[WOX3++]: YAB3.expr = reduced  //Oryza[WOX3++]: phenotype = Oryza[YAB3--].phenotype  Oryza[WOX3++]: leaf_lamina = twisted, leaf_margin = knotted, ligule = absent, auricle = absent//RNAi;  Oryza[WOX3++]: YAB3.expr = reduced  Hyp: WOX3 -s| YAB3 //from inducible WOX3 expression and DNA-protein interaction  Oryza[YAB3--]: KNOX.expr = ectopic, KNOX.expr = induced |
| 12426376 | Cell numbers and leaf development in Arabidopsis: a functional analysis of the STRUWWELPETER gene. | Autran D, Jonak C, Belcram K, Beemster GT, Kronenberger J, Grandjean O, Inze D, Traas J. | EMBO J. 2002 Nov 15;21(22):6036-49. | SPECIES: Arath  SUBJECT: leaf  SWP{At3g04740}=STREWWELPETER //lof;r;At3g04740;TDNA insertion mutant;WS ecotype  Arath[SWP--]:leaf.number=decreased  Arath[SWP--]:leaf.size=decreased //when no time is indicated, mature leaf is meant  Arath[SWP--]:root.size=decreased  Arath[SWP--]:embryo=normal  Arath[SWP--]:cotyledons.shape=lanceolate  Arath[SWP--]:cotelydons.color_intensity=increased  Arath[SWP--]:leaf[one_two].phyllochron=increased  Arath[SWP--]:leaf.lamina=elongated  Arath[SWP--]:leaf.lamina=serrate  Arath[SWP--]:leaf.epidermis_pavement_cell.size=increased  Arath[SWP--]:leaf.abaxial_epidermis_cell.size=increased  Arath[SWP--]:leaf_epidermis_stomatal_complex.size=increased  Arath[SWP--]:leaf_trichome.size=equal  Arath[SWP--]:leaf_trichome.branch.number=decreased  Arath[SWP--]:leaf.mesophyl_cell.size=increased  Arath[SWP--]:leaf.abaxial_epidermis_cell.number=decreased  Arath[SWP--]:leaf.cell.number=decreased  Arath[SWP--]:leaf.cell.size=increased  Arath[SWP--]:leaf.lamina.polarity=equal  Arath[SWP--]:petal.abaxial_epidermis_cell.size=equal  Arath[SWP--]:petal.abaxial_epidermis_cell.number=decreased  Arath[SWP--]:leaf.cell.ploidy=increased  Arath[SWP--]:leaf.size=decreased @d8_to_20  Arath[SWP--]:leaf.abaxial_epidermis_cell.size=decreased @d7_to_10  Arath[SWP--]:leaf.abaxial_epidermis_cell.size=decreased @d10_to_20  Arath[SWP--]:leaf.abaxial_epidermis_cell.number=decreased @d8_to_20  Arath[SWP--]:leaf.abaxial_epidermis_cell.cell_division.rate=decreased @d7_to_10  SWP{At3g04740}=STREWWELPETER //gof;D;At3g04740;35S CaMV promoter;Ws and Ler ecotypes  Arath[SWP++]:whole_plant.size=decreased  Arath[SWP++]:leaf.size=decreased  Arath[SWP++]:leaf.epidermis_pavement_cell.size=decreased  Arath[SWP++]:leaf.epidermis_pavement_cell.number=increased |
| 16684525 | Constitutive expression of abiotic stress-inducible hot pepper CaXTH3, which encodes a xyloglucan endotransglucosylase/hydrolase homolog, improves drought and salt tolerance in transgenic Arabidopsis plants. | Cho SK, Kim JE, Park JA, Eom TJ, Kim WT. | FEBS Lett. 2006 May 29;580(13):3136-44. Epub 2006 May 2. | SPECIES: Arabidopsis  SUBJECT: Abiotic_Stress  CaXTH1{DQ439860}=Xyloglycan_endotransglucosylase_hydrolase1_from_Capsicum_annuum  CaXTH2{DQ439861}=Xyloglycan_endotransglucosylase_hydrolase2_from_Capsicum_annuum  CaXTH3{DQ439862}=Xyloglycan_endotransglucosylase_hydrolase3_from_Capsicum_annuum  flat //PATO  drought_sensitivity //TO  salt_sensitivity //TO  sodium_chloride_regime //EO  root //PO  abscisic_acid_regime //EO  growth_rate //PATO  germination //PO  Arath[CaXTH3++]: leaf!=flat, cell.number++, cell.area--, drought_sensitivity--, salt_sensitivity-- //gof;promoter:CaMV 35S  @sodium_chloride_regime: Arath[CaXTH3++]: root.growth_rate++, germination++  @abscisic_acid_regime: Arath[CaXTH3++]: root.growth_rate++ |
| 15960617 | The transcription factor AtGRF5 and the transcription coactivator AN3 regulate cell proliferation in leaf primordia of Arabidopsis thaliana. | Horiguchi G, Kim GT, Tsukaya H. | Plant J. 2005 Jul;43(1):68-78. | SPECIES: Arath  SUBJECT: leaf  AN3{At5g28640}=angustifolia3  AtGRF5{At3g13960}=Growth_Regulating_factor5  Arath[AN3++]:leaf[one_two].leaf_lamina.area=increased, leaf[one_two].palisade_mesophyll_cell.area=equal, _  leaf[one_two].palisade_mesophyll_cell.number=increased, leaf_index=equal  //[Col0; GRF_Interacting_Factor1; AtGIF1; gof; 35Spromoter; sense]  Arath[AtGRF5++]:leaf[one_two].leaf_lamina.area=increased, leaf[one_two].palisade_mesophyll_cell.area=equal, _  leaf[one_two].palisade_mesophyll_cell.number=increased, leaf_index=equal  //[Col0; gof; 35Spromoter; sense]  Arath[AtGRF5--]:leaf.area=narrow, leaf[one_two].palisade_mesophyll_cell.number=decreased  //[Col0; lof; genuine; T-DNA_insertion; first_intron;atgrf5_1]  Arath[AN3_4--]:leaf.area=narrow, leaf.lamina.width=decreased, leaf.lamina.length=decreased, _  leaf_index=increased, leaf.petiole.length=decreased, leaf[one_two].palisade_mesophyll_cell.number=decreased, _  leaf.number=decreased, leaf[one_two].palisade_mesophyll_cell.area=increased, flower.petal.width=decreased  //[Col0; lof; genuine; 6_base_deletion] |
| 16669771 | Mechanism of leaf-shape determination. | Tsukaya H. | Annu Rev Plant Biol. 2006;57:477-96. | SPECIES: Arabidopsis  SUBJECT: leaf  leaf //PO  length //PATO  widht //PATO  epidermal_pavement_cell //PO  epidermal_meristemoid_cell //PO  leaf_margin //PO  //serrate //PATO  serrate.number  serrate.depth  //lobate.number  //lobate.depth  depth // PATO  //lobate //PATO  //entire //PATO  number//PATO  increased //PATO  decreased //PATO  size //PATO  curvature //PATO  leaf.epidermis_pavement_cell.number  leaf.epidermis_pavement_cell.size  leaf_lamina //PO  leaf_lamina.length  leaf_lamina.width  leaf_lamina.flat  leaf_lamina.curvature  petiole // PO  leaf.petiole  leaf.petiole.length  flat //PATO  //!=flat //PATO  palisade_mesophyll_cell //PO  leaf_vascular_system //PO  abnormal //PATO  vascular //PO  ROT3{ATxxxx}=rotundifolia3  ROT4{ATxxxx}=rotundifolia4  AN{ATxxxx}=angustifolia  AN3{ATxxxx}=angustifolia3  SPIKE1{ATxxxx}=spike1  AS{ATxxxx}=asymmetric_leaves  SE{ATxxxx}=serrated  KRP2{At3g50630}=Kip_Related_Protein2  JAG{ATxxxx}=jagged  PFL2{ATxxxx}=pointed_first_leaf  DRL1{ATxxxx}=deformed_roots_and_leaves1  SWP{ATxxxx}=struwwelpeter  JAW{ATxxxx}=jaw_miRNA_locus  ER{ATxxxx}=erecta  leaf=)leaf_lamina  Arath[ROT3--]:leaf_lamina.length=decreased, leaf_epidermis_pavement_cell.size=decreased //rot3_1  Arath[ROT4--]:leaf_lamina.length=decreased, leaf_epidermis_pavement_cell.number=decreased //rot4_1D  Arath[AN--]:leaf_lamina.width=decreased, leaf_epidermis_pavement_cell.size=decreased //an  Arath[AN3--]:leaf_lamina.width=decreased, leaf_epidermis_pavement_cell.number=decreased,leaf_margin=entire //an3  Arath[AN3_4--]:leaf_lamina.width=decreased, leaf.palisade_mesophyll_cell.number=decreased,leaf.palisade_mesophyll_cell.size=increased  //an3_4  Arath[AN3_ATGIF1--]:leaf_lamina.width=decreased, leaf.epidermis_pavement_cell.number=decreased, leaf.epidermis_pavement_cell.size=increased //an3/atgif1  Arath[SPIKE1--]:leaf_lamina.width=decreased, leaf.epidermis_pavement_cell.size=decreased //spk1  Arath[AS1--]:leaf_margin=serrate, serrate.depth=increased, serrate.number=decreased //as1  Arath[AS2--]:leaf_margin=serrate, serrate.depth=increased, serrate.number=decreased //as2  Arath[SE--]:leaf_margin=serrate, serrate.depth=increased, serrate.number=increased //se  Arath[KRP2++]:leaf_lamina.length=decreased, leaf_lamina.width=decreased, leaf.palisade_mesophyll_cell.size=increased,leaf.palisade_mesophyll_cell.number=decreased, _  leaf.epidermis_pavement_cell.size=increased,leaf.epidermis_pavement_cell.number=decreased, leaf_margin=serrate, serrate.depth=increased  //KRP2OE  Arath[JAG--]:leaf_margin=serrate, serrate.depth=increased //jag  Arath[PFL2--]:leaf_margin=entire //pfl2  Arath[DRL1--]:leaf_margin=entire //drl1  Arath[SWP--]:leaf_margin=entire //drl1  Arath[JAW++]:leaf_lamina!=flat,leaf_margin=serrate //JAWOE  Arath[ER--]:leaf_lamina.length=decreased, leaf.palisade_mesophyll_cell.size=increased,leaf.palisade_mesophyll_cell.number=decreased, _  leaf_margin=entire //er_102 |
| 16916932 | PEAPOD regulates lamina size and curvature in Arabidopsis. | White DW. | Proc Natl Acad Sci U S A. 2006 Aug 29;103(35):13238-43. Epub 2006 Aug 17. | SPECIES : Arath //Ler ecotype  SUBJECT : Leaf_development  PPD = PEAPOD //PPD-- is a 60kb deletion from At4g14700 to At4g14760  PPD1{At4g14713}  PPD2{At4g14720}  (PPD1, PPD2) (= PPD  PPD1 =h PPD2  Arath[PPD--]: cotelydon.vascular_system.areole.number = increased  Arath[PPD--]: cotelydon.lamina.size = increased  Arath[PPD--]: cotelydon.lamina.shape = flat  Arath[PPD--]: leaf_lamina.size = increased  Arath[PPD--]: leaf_lamina.shape = domed //prolonged DMC proliferation  Arath[PPD--]: fruit.length = decreased, fruit.width = increased //flattened  Arath[PPD--]: trichome.branch = decreased //2 in stead of 3 or 4  Arath[PPD++]: leaf_lamina.size = decreased //early arrest of DMC proliferation  //Arath[PPD--][PPD1++]: (whole_plant *except* trichome.branch) = equal  Arath[PPD--][PPD1++]: trichome.branch = decreased  Arath[PPD--]: leaf_abaxial_epidermis.stomata.number = increased // 137%  //Arath[PPD--]: foci of meristemoid cells that keep on dividing longer than WT (stop at d20 in stead of 12) |
| 12953103 | The Arabidopsis auxin-inducible gene ARGOS controls lateral organ size. | Hu Y, Xie Q, Chua NH. | Plant Cell. 2003 Sep;15(9):1951-61. | SPECIES: Arabidopsis  SUBJECT: leaf  ARGOS{At3g59900}=Auxin_Regulated_Gene_Involved_in_Organ_Size  Arath[ARGOS--]:leaf_lamina.length=decreased, leaf_lamina.width=decreased, leaf.palisade_mesophyll_cell.size=decreased,leaf.palisade_mesophyll_cell.number=decreased, leaf.petiole.length=decreased  //ARGOS_antisense_OE  Arath[ARGOS++]:leaf_lamina.length=increased,leaf_lamina.width=increased,leaf.palisade_mesophyll_cell.size=increased,leaf.palisade_mesophyll_cell.number=increased,leaf.petiole.length=increased //ARGOSOE |
| 17209125 | Arabidopsis WEE1 kinase controls cell cycle arrest in response to activation of the DNA integrity checkpoint. | De Schutter K, Joubes J, Cools T, Verkest A, Corellou F, Babiychuk E, Van Der Schueren E, Beeckman T, Kushnir S, Inze D, De Veylder L. | Plant Cell. 2007 Jan;19(1):211-25. Epub 2007 Jan 5. | SPECIES: Arabidopsis  SUBJECT: leaf  WEE1{At1g02970} = WEE1_Kinase  Arath[WEE1--]: leaf_lamina.length = equal //wee1TDNA_insertion  Arath[WEE1--]: leaf_lamina.width = equal //wee1TDNA_insertion  Arath[WEE1--]: leaf.epidermis_pavement_cell.size = equal //wee1TDNA_insertion  Arath[WEE1--]: leaf.epidermis_pavement_cell.number = equal //wee1TDNA_insertion |
| 17286797 | Sugar-inducible expression of the nucleolin-1 gene of Arabidopsis thaliana and its role in ribosome synthesis, growth and development. | Kojima H, Suzuki T, Kato T, Enomoto K, Sato S, Kato T, Tabata S, Saez-Vasquez J, Echeverria M, Nakagawa T, Ishiguro S, Nakamura K. | Plant J. 2007 Mar;49(6):1053-63. Epub 2007 Feb 7. | SPECIES: Arabidopsis  SUBJECT: leaf  AtNuc_L1{At1g48920}=Nucleolin1  Arath[AtNuc_L1--]:leaf_lamina.width=decreased, leaf_vascular_system=abnormal |
| 16990135 | Activation of glucosidase via stress-induced polymerization rapidly increases active pools of abscisic acid. | Lee KH, Piao HL, Kim HY, Choi SM, Jiang F, Hartung W, Hwang I, Kwak JM, Lee IJ, Hwang I. | Cell. 2006 Sep 22;126(6):1109-20. | SPECIES: Arabidopsis  SUBJECT: abiotic_stress  AtBG1{AT1G45130}=beta-glucosidase1  AtBG2=beta-glucosidase2  leaf_color //TO  abscisic_acid_concentration //TO  dark //PATO  yellow //PATO  closure //PATO  stomata //PO  Arath[AtBG1++]: salt_sensitivity-- //gof; promoter: CaMV 35S  Arath[AtBG1--]: whole_plant.size--, leaf_color=yellow, abscisic_acid_concentration--, drought_sensitivity++ //lof; genuine; T-DNA insertion mutant  @dark: Arath[AtBG1]: stomata.closure=abnormal  @absisic_acid_regime: Arath[AtBG1--]: whole_plant.size=equal, leaf_color=equal,abscisic_acid_concentration=equal, drought_sensitivity=equal  @abisic_acid_regime: @dark: Arath[AtBG1]: stomata.closure=equal  Arath[AtBG2--]: whole_plant.size--, leaf_color=yellow,abscisic_acid_concentration--, drought_sensitivity++ //lof; genuine; T-DNA insertion; same phenotype as AtBG1-- but weaker mutant  @dark: Arath[AtBG2]: stomata.closure=abnormal |
| 10639184 | Plant organ size control: AINTEGUMENTA regulates growth and cell numbers during organogenesis. | Mizukami Y, Fischer RL. | Proc Natl Acad Sci U S A. 2000 Jan 18;97(2):942-7. | ANT{Atxxxx}=Aintegumenta  Arath[ANT++]:leaf_lamina.length=increased, leaf.epidermis_pavement_cell.number=increased //ANTOE  Arath[ANT--]:leaf_lamina.length=decreased, leaf_lamina.width=decreased, leaf.epidermis_pavement_cell.number=decreased, leaf.epidermis_pavement_cell.size=increased //ant_1 |
| 17098812 | A membrane-bound NAC transcription factor regulates cell division in Arabidopsis. | Kim YS, Kim SG, Park JE, Park HY, Lim MH, Chua NH, Park CM. | Plant Cell. 2006 Nov;18(11):3132-44. Epub 2006 Nov 10. | SPECIES: Arabidopsis  SUBJECT: leaf  NTM1{At4g01540} = NAC_with_transmembrane_motif  Arath[NTM1++]: leaf_margin = serrate //ntm1_D  Arath[NTM1++]: serrate.depth = increased //ntm1_D  Arath[NTM1++]: leaf_lamina.length = decreased //ntm1_D  Arath[NTM1++]: leaf_lamina.width = decreased //ntm1_D  Arath[NTM1++]: leaf.epidermis_pavement_cell.number = decreased //ntm1_D  Arath[NTM1++]: leaf.epidermis_pavement_cell.size = increased //ntm1_D |
| 16805736 | Impaired sucrose induction1 encodes a conserved plant-specific protein that couples carbohydrate availability to gene expression and plant growth. | Rook F, Corke F, Baier M, Holman R, May AG, Bevan MW. | Plant J. 2006 Jun;46(6):1045-58. | SPECIES: Arabidopsis  SUBJECT: leaf  ISI1{At4g27750}=Impaired_Sucrose_Induction1  Arath[ISI1--]:leaf_lamina.length=decreased |
| 16617092 | Mutations in the microRNA complementarity site of the INCURVATA4 gene perturb meristem function and adaxialize lateral organs in arabidopsis. | Ochando I, Jover-Gil S, Ripoll JJ, Candela H, Vera A, Ponce MR, Martinez-Laborda A, Micol JL. | Plant Physiol. 2006 Jun;141(2):607-19. Epub 2006 Apr 14. | SPECIES: Arabidopsis  SUBJECT: leaf  ICU4{At1g52150}=Incurvata4  Arath[ICU4++]: leaf_lamina.curvature=increased, leaf.epidermis_pavement_cell.size=decreased //icu4_1,icu4_2, gain_of_function  Arath[ICU4--]: leaf_lamina.curvature=normal //ICU4_RNAiOE, icu4_3, icu4_4 |
| 15834008 | Arabidopsis CBF3/DREB1A and ABF3 in transgenic rice increased tolerance to abiotic stress without stunting growth. | Oh SJ, Song SI, Kim YS, Jang HJ, Kim SY, Kim M, Kim YK, Nahm BH, Kim JK. | Plant Physiol. 2005 May;138(1):341-51. Epub 2005 Apr 15. | SPECIES: Orysa_sativa  SUBJECT: abiotic_stress  water_content //PATO  ABF3{AT4G34000}=ABSCISIC_ACID_RESPONSIVE_ELEMENTS-BINDING_FACTOR_3  DREB1A{AT4G25480}=DEHYDRATION_RESPONSE_ELEMENT_B1A  Orysa[ABF3++]:growth_rate=equal,germination=equal, drought_sensitivity--, cold_tolerance++ //gof, promoter maize UBI1 can be promoters not added as ubu1:abf3++?  Orysa[DREB1A++]:growth_rate=equal,germination=equal, drought_sensitivity--, cold_tolerance++ //gof, promoter maize UBI1  @water_content.low:Orysa[ABF3++]:growth_rate=increased  @water_content.low:Orysa[DREB1A++]:growth_rate=increased |
| 11449057 | Functional analysis of cyclin-dependent kinase inhibitors of Arabidopsis. | De Veylder L, Beeckman T, Beemster GT, Krols L, Terras F, Landrieu I, van der Schueren E, Maes S, Naudts M, Inze D. | Plant Cell. 2001 Jul;13(7):1653-68. | // THIS ANNOTATION IS NOT FINISHED //  SPECIES: Arabidopsis //Col-0  SUBJECT: cell cycle cire genes  // viability //PATO  // viable //PATO  // growth timing quality //PATO  ////Arath[KRP1++]  // [gof;D+;transgene;35S]  leaf=)leaf[five]  leaf=)leaf[one_two]  Arath[KRP2++]:leaf.width=decrease  Arath[KRP2++]:leaf_margin.serrate=increased  Arath[KRP2++]:leaf.number=equal  Arath[KRP2++]:leaf[five].leaf_lamina_adaxial_epidermis.cell.size=increased  Arath[KRP2++]:leaf[five].palisade_mesophyll.cell.area=increased  Arath[KRP2++]:leaf[five].spongy_mesophyll.cell.area=increased  Arath[KRP2++]:leaf[five].leaf_lamina_abaxial_epidermis.cell.area=increased  Arath[KRP2++]:leaf[five].trichome.area=equal  Arath[KRP2++]:leaf[five].stomatal_complex.area=equal  Arath[KRP2++]:leaf[five].thickness=increased  Arath[KRP2++]:leaf[one_two][@maturity].area=decreased  Arath[KRP2++]:leaf[one_two].growth_timing_quality=equal  //Clearly, the duration of expansion was unaffected by the transgene.  ////Arath[KRP2++]:leaf[one_two].cell.number=  // at maturity // how to express this? @T=maturity or [@T=maturity], where to put it?  //From day 5 until day 21 after sowing, leaves of transgenic and wild-type plants  //were harvested and leaf size and number, size of the abaxial epidermal cells, and  //stomatal index were determined (see Methods).  // [gof;D+;transgene;35S] //mRNA and protein levels were confirmed higher than in wt  ////Arath[KRP3++]  // [gof;D+;transgene;35S]  HYP:Arath[KRP4++]:viability=viable // several independent transformations yielded no transgenics  // [gof;D+;transgene;35S] |
| 11489171 | Overexpression of DWARF4 in the brassinosteroid biosynthetic pathway results in increased vegetative growth and seed yield in Arabidopsis. | Choe S, Fujioka S, Noguchi T, Takatsuto S, Yoshida S, Feldmann KA. | Plant J. 2001 Jun;26(6):573-82. | SPECIES: Arath  SUBJECT: leaf  DWF4{At3g50660}=DWARF4  Arath[DWF4++]:leaf.petiole.length=increased, leaf.lamina.length=increased, leaf.number=equal, _  inflorescence.length=increased, inflorescence_branch.number=increased, fruit.number=increased, _  seed.number=increased  //[WS-2; gof; 35Spromoter; sense;AOD4] |
| 16709191 | Maize DBF1-interactor protein 1 containing an R3H domain is a potential regulator of DBF1 activity in stress responses. | Saleh A, Lumbreras V, Lopez C, Dominguez-Puigjaner E, Kizis D, Pages M. | Plant J. 2006 Jun;46(5):747-57. | SPECIES: Arabidopsis  SUBJECT: Abiotic_stress  DBF1{AAM80486}=DRE_binding_factor_1  chlorosis  vigour  plant_survivability //TO  Zeama[DBF1++]: growth_rate=decreased // gof, promoter: CaMV 35S; it should be possible to distinguish between these two constructs  Zeama[DBF1++]: growth_rate=equal // gof, promoter: RD29A=stress inducible  @sodium_chloride_regime:Zeama[DBF1++]: germination=increased, growth_rate=increased, _  chlorosis=decreased, vigour=increased, leaf.number=increased // could not find Ontology term for vigour or chlorosis  @water_content.low:Zeama[DBF1++]:plant_survivability=increased //should think of other statement for applying drought stress |
| 16514015 | Interplay between Arabidopsis activating factors E2Fb and E2Fa in cell cycle progression and development. | Sozzani R, Maggio C, Varotto S, Canova S, Bergounioux C, Albani D, Cella R. | Plant Physiol. 2006 Apr;140(4):1355-66. Epub 2006 Mar 2. | SPECIES: Arath  SUBJECT: E2F  E2Fb{}  Arath[E2Fb++]: primary_root.length = decreased //3-fold  Arath[E2Fb++]: root_hair.density = increased, lateral_root_primordium.density = increased, hypocotyl.length = decreased  Arath[E2Fb++]: leaf[one_two].trichome = absent  Arath[E2Fb++]: cotelydon.epidermal_cell.size = decreased, cotelydon.epidermal_cell.number = increased, cotelydon.size = equal//in fact a small increase  Arath[E2Fa++]: cotelydon.epidermal_cell.size = decreased, cotelydon.epidermal_cell.number = increased, cotelydon.size = equal//in fact a small increase |
| 16332447 | Vascular development: the long and winding road. | Sieburth LE, Deyholos MK. | Curr Opin Plant Biol. 2006 Feb;9(1):48-54. Epub 2005 Dec 5. | SPECIES: Arath  SUBJECT: Leaf_Development  sfc{AT5G13300}=SCARFACE  van3{AT5G13300}=VASCULAR_NETWORK_DEFECTIVE_3  sfc=van3  tkv{AT5G19530}=thickvein  acl5{AT5G19530}=ACAULIS_5  acl5=tkv  cov1{AT2G20120}=CONTINUOUS_VASCULAR_RING  //Arath[acl5--]:leaf.vein_thickness=increased  //Arath[acl5--]:stem.vein_thickness=increased  //Arath[cov1--]:@stem xylem.amount=increased  //Arath[cov1--]:@stem phloem.amount=increased  ////Arath[cov1--]:@stem_base vascular_bundle.number=increased |
| 17351056 | E2F Regulates FASCIATA1, a Chromatin Assembly Gene whose Loss Switches on the Endocycle and Activates Gene Expression by Changing the Epigenetic Status. | Ramirez-Parra E, Gutierrez C. | Plant Physiol. 2007 Mar 9;. | SPECIES: Arath  SUBJECT: leaf  FAS1{AT1G65470}=FASCIATA1_Chromatin_Assembly_Factor-1_CAF-1_p150_subunit  Arath[FAS1--]:leaf.shape=dentate,leaf.shape=small,leaf.shape=narrow //fas1-4 mutant _  leaf_adaxial_epidermis=abnormal//presence of very large cells, _  leaf_mesophyll=abnormal,leaf_adaxial_epidermis.cell=large//fas1-4 adaxial leaf epidermis contained approximately 3.5-fold fewer, but approximately 2-fold larger, cells than the wild type, _  leaf_adaxial_epidermis.cell.number--,leaf_trichome.branch.number++//45% of fas1-4 trichomes developed more than four branches, in clear contrast to the wild-type situation, where approximately 3% of trichomes contain more than three branches, _  cotyledon.ploidy++//increased ploidy phenotype was consistently observed in cotyledons and even flowers, which do not normally have a significant proportion of >4C nuclei |
| 16418486 | A systematic search for downstream mediators of tumor suppressor function of p53 reveals a major role of BTG2 in suppression of Ras-induced transformation. | Boiko AD, Porteous S, Razorenova OV, Krivokrysenko VI, Williams BR, Gudkov AV. | Genes Dev 2006 Jan 15;20(2):236-52 | NF-kappa_B -> BTG2  Arf -> BTG2  p53 -> BTG2  BTG2 -s| cyclin_D1 |
| 16054292 | Gastrin and cancer: a review. | Ferrand A, Wang TC. | Cancer Lett 2006 Jul 8;238(1):15-29 | //gastrin binds and activates CCK2R  gastrin <-> CCK2R  gastrin -> CCK2R  gastrin -> (P -> Shc)  Src -> (P -> Shc)  Shc[P] <-> (Grb2,Sos)  (Shc[P], Grb2/Sos) -> (P -> Ras)  Ras[P] -> (P -> Raf)  Raf[P] -> (P -> MEK)  MEK[P] -> (P -> ERK)  gastrin -s> Reg_1 |
| 17128210 | Gastrin - active participant or bystander in gastric carcinogenesis? | Watson SA, Grabowska AM, El-Zaatari M, Takhar A. | Nat Rev Cancer 2006 Dec;6(12):936-46 | gastrin_receptor_activity -s> anti_apoptotic_proteins  gastrin_receptor_activity -> _1_phosphatidylinositol_3_kinase_activity  _1_phosphatidylinositol_3_kinase_activity -> protein_kinase_B_binding  gastrin_receptor_activity -s> protein_kinase_B_binding  gastrin_receptor_activity -s> protein_kinase_B_binding  gastrin_receptor_activity -> (P->BAD)  gastrin_receptor_activity -s> AKT  protein_kinase_B_signaling_cascade ->(P -> BAD)  gastrin_receptor_activity -s> anti_apoptotic_proteon_x_linked_inhibitor_of_apoptosis_XIAP  gastrin_receptor_activity  _1_phosphatidylinositol_3_kinase_activity -> protein_kinase_B_binding  protein_kinase_B_signaling_cascade -> (P-> BAD)  gastrin -> gastric_acid_secretion |
| 17239973 | Characteristics of gastrin controlled ECL cell specific gene expression. | Friis-Hansen L, Schjerling CK, de la Cour CD, Hakanson R, Rehfeld JF. | Regul Pept 2007 May 3;140(3):153-61 | defender_against_cell_death_1 -| apoptosis  gastrin -> CD63_antigen  gastrin -s> defender_against_cell_death_1  defender_against_cell_death_1 -t> defender_against_cell_death_1[@loc=cell_membrane]  gastrin -s> BCL2_associated_X_protein_gene  gastrin -> G_protein_coupled_receptor  G_protein_coupled_receptor -> calbindin_1  calbindin_1 -> (P -> neudesin) |
| 17490981 | Dietary carbohydrate modification induces alterations in gene expression in abdominal subcutaneous adipose tissue in persons with the metabolic syndrome: the FUNGENUT Study. | Kallio P, Kolehmainen M, Laaksonen DE, Kekalainen J, Salopuro T, Sivenius K, Pulkkinen L, Mykkanen HM, Niskanen L, Uusitupa M, Poutanen KS. | Am J Clin Nutr 2007 May;85(5):1417-27 | SPECIES: human  IGFPB_5 ~> IGF_I  IGFPB_5 ~> IGF_II  HYP: IGFBP_3 = IGFBP_5  hormone_sensitive_lipase -> adipocyte_hypertrophy |
| 17028200 | Regulatory T cell-mediated suppression: potential role of ICER. | Bodor J, Fehervari Z, Diamond B, Sakaguchi S. | J Leukoc Biol 2007 Jan;81(1):161-7 | Species: human  ICER -|CBP  ICER -|CREB  CREB <-> CBP  NFAT <-> CBP  NFkB <-> CBP  //CREB -s> CREB.DNA.CRE  CBP <-> STAT  CBP <-> Smad  transforming_growth_factor_beta_receptor_activity ->Smad  MAPK -> (P->ICER) |
| 17496911 | G protein regulation of MAPK networks. | Goldsmith ZG, Dhanasekaran DN. | Oncogene 2007 May 14;26(22):3122-42 | SPECIES: Homo_sapiens  SUBJECT: MAPK networks  Gbeta <-> Ggamma  Gs -> positive_regulation_of_adenylate_cyclase_activity  cAMP_biosynthetic_process{high}  cAMP -> cAMP_dependent_protein_kinase_activity  cAMP_dependent_protein_kinase_activity -> EPAC  EPAC -> (P -> Rap_1)  Rap_1 -> B_Raf  PKA -> (P -> C_Raf) |
| 17579082 | Negative regulation of TLR responses by the neuropeptide CGRP is mediated by the transcriptional repressor ICER. | Harzenetter MD, Novotny AR, Gais P, Molina CA, Altmayr F, Holzmann B. | J Immunol 2007 Jul 1;179(1):607-15 | TLR -> TNF_alpha  TLF -> CCL4  Calcitonin_gene_related_peptide = CGRP  CGRP -s| TNF_alpha  CGRP -> ICER  CGRP -| CCL4  cAMP_biosynthetic_process -> ICER  ICER -| TNF_promoter  LPS -> TNF_promoter  ICER -s| TNF_alpha  CRLR = calcitonin_receptor_like_receptor  CRLR <-> RAMP2  CRLR <-> RAMP3 |
| 17565599 | TORC-SIK cascade regulates CREB activity through the basic leucine zipper domain. | Takemori H, Kajimura J, Okamoto M. | FEBS J 2007 Jul;274(13):3202-9 | TORC -> CREB  SIK1 -| CREB  PKA -> (P ->CREB)  PKA -> (P ->SIK1)  SIK1[P] -t> SIK1[P][@loc=cytoplasm]  CREB -s> CYP11A1  SIK1 -s| CYP11A1  SIK1 -s| StAR  PKA -> CYP11A1  SIK1 -> (P -> TORC) |
| 17496915 | Scaffold proteins of MAP-kinase modules. | Dhanasekaran DN, Kashef K, Lee CM, Xu H, Reddy EP. | Oncogene 2007 May 14;26(22):3185-202 | species: human  SUBJECT: Scaffold proteins of MAP-Kinase modules  MAP_kinase_kinase_kinase_kinase_activity -> MAP_kinase_kinase_kinase_activity  MAP_kinase_kinase_kinase_activity -> MAP_kinase_kinase_activity  MAP_kinase_kinase_activity -> MAP_kinase_activity |
| 17426018 | A tandem affinity purification-based technology platform to study the cell cycle interactome in Arabidopsis thaliana. | Van Leene J, Stals H, Eeckhout D, Persiau G, Van De Slijke E, Van Isterdael G, De Clercq A, Bonnet E, Laukens K, Remmerie N, Henderickx K, De Vijlder T, Abdelkrim A, Pharazyn A, Van Onckelen H, Inze D, Witters E, De Jaeger G. | Mol Cell Proteomics 2007 Jul;6(7):1226-38 | CDKA;1 <-> (CKS1,CKS2, KRP4, CDKE;1, CYCD4;1, CYCD4;2, KRP6)  CDKA;1 <-> (At2g20580, At5g23540, At1g64520, At5g40460, At1g23190, At4g28470, At1g10690, At3g49240, At2g28000, At3g17020, At1g78900, At3g55000)  CDKB1;1 <-> (CKS2, At2g28000, At1g64520)  CDKD;2 <-> (CYCH;1, At5g08690, At5g08670, At4g30820, At4g16143)  CDKF;1 <-> (At3g16270, CDKG;2, CDKD;2)  CKS1 <-> (CDKA;1, CDKB1;1, CYCA3;4, At4g14310,CDKB2;2, CYCD2;1,Arath05g16630,At3g53880, CDKB2;1)  CYCD3;1 <-> (CDKA;1, CKS2, KRP6, At5g02220) |
| 17287251 | R1R2R3-Myb proteins positively regulate cytokinesis through activation of KNOLLE transcription in Arabidopsis thaliana. | Haga N, Kato K, Murase M, Araki S, Kubo M, Demura T, Suzuki K, Muller I, Voss U, Jurgens G, Ito M. | Development 2007 Mar;134(6):1101-10 | Arath[MYB3R1--][MYB3R4--]:CYCB2;1--, KNOLLE--, CDC20_1--  KNOLLE.promoter =) MSA_element  CDC20_1.promoter =) MSA_element  CYCB2;1.promoter =) MSA_element |
| 12535341 | Diverse RNA viruses elicit the expression of common sets of genes in susceptible Arabidopsis thaliana plants. | Whitham SA, Quan S, Chang HS, Cooper B, Estes B, Zhu T, Wang X, Hou YM. | Plant J 2003 Jan;33(2):271-83 | SPECIES: Arabidopsis  ORMV = oilseed_rape_mosaic_tobamovirus  PVX = potato_virus_X  TuMV = turnip_mosaic_potyvirus  TVCV = turnip_vein_clearing_tobamovirus  CMV = cucumber_mosaic_cucumovirus  DAI = days_after_inoculation  salicylic_acid (= signaling_molecules  defence_response ->(PAD3, PAD4, PR1, PR5, BG2, Cf_2).expr  heat_shock_treatment -> (HSP101, HSP83, HSP70, HSP23_6, HSP17_6A, HSP17_4).expr  (BG2, PR1, PR5, PAD4, PAD3) (= salicylic_acid_mediated_signaling_pathway_  //(Bowling et al., 1994; Jirage et al., 1999; Ward et al., 1991; Zhou et al., 1999).  Arath [ORMV_virus_infection] : PAD4.expr=induced @(_2DAI, _5DAI)  Arath [PVX_virus_infection] : PAD4.expr=induced @(_2DAI, _5DAI)  Arath [CMV_virus_infection] : PAD4.expr=induced @(_2DAI, _5DAI)  Arath [TuMV_virus_infection] : PAD4.expr=induced @_5DAI  Arath [TVCV_virus_infection] : PAD4.expr=induced @_5DAI  Arath [ORMV_virus_infection] : BG2.expr=induced @(_2DAI, _4DAI, _5DAI)  Arath [PVX_virus_infection] : BG2.expr=induced @ (_2DAI, _4DAI, _5DAI)  Arath [CMV_virus_infection] : BG2.expr=induced @(_2DAI, _4DAI, _5DAI)  Arath [TuMV_virus_infection] : BG2.expr=induced @(_2DAI, _4DAI, _5DAI)  Arath [TVCV_virus_infection] : BG2.expr=induced @(_2DAI, _4DAI, _5DAI)  Arath [PVX_virus_infection] : HSP101.expr=induced @(_2DAI, _4DAI, _5DAI)  Arath [ORMV_virus_infection] : HSP101.expr=induced @_1DAI  Arath [TVCV_virus_infection] : HSP101.expr=induced @_1DAI  Arath [CMV_virus_infection] : HSP101.expr=none  Arath [TuMV_virus_infection] : HSP101.expr=none |
| 15728340 | Salicylic acid-dependent expression of host genes in compatible Arabidopsis-virus interactions. | Huang Z, Yeakley JM, Garcia EW, Holdridge JD, Fan JB, Whitham SA. | Plant Physiol 2005 Mar;137(3):1147-59 | SPECIES: Arabidopsis  mut = mutation  NahG = transgene_encoding_bacterial_salicylate_hydroxylase  PR_genes = pathogenesis_related_genes  CMV_Y = cucumber_mosaic_cucumovirus  ORMV = oilseed_rape_mosaic_tobamovirus  PDF1_2 = biomarker_for_jasmonic_acid_and_ethylene_mediated_signaling_pathways  (salicylic_acid, jasmonic_acid, ethylene) (= signaling_molecules  (salicylic_acid, jasmonic_acid, ethylene) ~> defense_response_to_pathogen  (EDS1, PAD4, NPR1) (= salicylic_acid_mediated_signaling_pathway  salicylic_acid -> (EDS1, PAD4, NPR1).expr  (EDS5, SID2) ~> salicylic_acid_biosynthetic_process  Arath [NahG] : NahG -| salicylic_acid_biosynthetic_process  NahG -> (salicylic_acid -t> catechol)  //MineMap problem//// e[many] defence_response_to_pathogen : cathechol -| defence_response_to_pathogen  Arath [NPR1,mut] : npr1-| salicylic_acid_mediated_defence_response  salicylic_acid -> NPR1 -t> NPR1 [@nucleus]  NPR1 [@nucleus] + TGA -t> NPR1_TGA //NPR1^TGA  NPR1_TGA -s> PR_genes  (JAR1, COI1) (= jasmonic_acid_mediated_signaling_pathway  (JAR1, COI1) (= ethylene_mediated_signaling_pathway  (JAR1, COI1) ~> jasmonic_acid_biosynthetic_process  (JAR1, COI1) -s> PDF1_2  EIN2 (= jasmonic_acid_mediated_signaling_pathway  EIN2 -> PDF1_2.expr ++  //RESULTS  (PR_1, PR_5, Bgl2) (= biomarkers_for_salicylic_acid_mediated_signaling_pathway  Arath [NahG] [CMV_Y_virus_infection] : PR_1.expr --  Arath [NahG] [CMV_Y_virus_infection] : Bgl2.expr --  Arath [NahG] [CMV_Y_virus_infection] : PR_5.expr --  Arath [NahG] [ORMV_virus_infection] : PR_1.expr --  Arath [NahG] [ORMV_virus_infection] : Bgl2.expr --  Arath [NahG] [ORMV_virus_infection] : PR_5.expr --  PR_1 ~> (EDS5, NPR1).expr  At2g14560 ~> (EDS5, NPR1).expr    Arath [EDS5,mut] [CMV_Y_virus_infection] : PR_1.expr --  Arath [EDS5,mut] [CMV_Y_virus_infection] : At2g14560.expr --  Arath [EDS5,mut] [ORMV_virus_infection] : PR_1.expr --  Arath [EDS5,mut] [ORMV_virus_infection] : At2g14560.expr --  Arath [NPR1,mut] [ORMV_virus_infection] : PR_1.expr --  Arath [NPR1,mut] [ORMV_virus_infection] : At2g14560.expr --  Arath [NPR1,mut] [CMV_Y_virus_infection] : PR_1.expr --  Arath [NPR1,mut] [CMV_Y_virus_infection] : At2g14560.expr --  Arath [JAR1,mut] [CMV_Y_virus_infection] : PR_1.expr ++  Arath [JAR1,mut] [ORMV_virus_infection] : PR_1.expr ++  Arath [EIN2,mut] [CMV_Y_virus_infection] : PR_1.expr ++  Arath [EIN2,mut] [ORMV_virus_infection] : PR_1.expr ++  (JAR1, EIN1) -| PR_1.expr  Arath [NahG] [ORMV_virus_infection] : PDF1.expr=low  Arath [NahG] [CMV_Y_virus_infection] : PDF1.expr=low  Arath [SID2,mut] [ORMV_virus_infection] : PDF1.expr=low  Arath [SID2,mut] [CMV_Y_virus_infection] : PDF1.expr=low |
| 16169957 | Cauliflower mosaic virus, a compatible pathogen of Arabidopsis, engages three distinct defense-signaling pathways and activates rapid systemic generation of reactive oxygen species. | Love AJ, Yun BW, Laval V, Loake GJ, Milner JJ. | Plant Physiol 2005 Oct;139(2):935-48 | SPECIES: Arabidopsis  ROS = reactive_oxygen_species  CaMV = cauliflower_mosaic_virus  RSR = rapid_systemic_response  dmut = double_mutation  (PR_1, Bgl_2, PR_5) = biomarkers_for_salicylic_acid_mediated_signaling_pathway  GST1 = biomarker_for_responce_to_ROS  PDF1_2 = biomarker_for_jasmonic_acid_and_ethylene_mediated_signaling_pathways  NADPH = generator_of_ROS  (salicylic_acid, jasmonic_acid, ethylene) (= signaling_molecules  (salicylic_acid, jasmonic_acid, ethylene) ~> defence_response__incompatible_interaction  dpi = dots_per_inch  Antimycin_A = inducer_of_resistance  NahG = transgene_encoding_bacterial_salicylate_hydroxylase  rbohDF = NADPH_double_oxidase_mutant  //Results  salicylic_acid -> (PR1, PR2, PR5).expr  ROS -> GST1.expr  Arath [CaMV_virus_infection] : (PR_1,PR_2, PR_5).expr=low @_5dpi  Arath [CaMV_virus_infection] : (PR_1,PR_2, PR_5).expr=high @_8dpi  Arath [CaMV_virus_infection] : GST1.expr=high @_2dpi  Arath [CaMV_virus_infection] : (PR_1,PR_2, PR_5).expr=low_until @_8dpi  Arath [CaMV_virus_infection] : (PR_1,PR_2, PR_5).expr=high_after @_8dpi  Arath [CaMV_virus_infection] : GST1.expr=high @_2dpi  Antimycin_A -> mitochondrial_electron_transport //transport through the Aox_pathway  Arath [CaMV_virus_infection] [Antimycin_A_inoculation] : ROS_generation=present //only when virus and antimycin A were coinoculated  Arath [CaMV_virus_infection] [Antimycin_A_inoculation] : virus_defence=present //only when virus and antimycin A were coinoculated  Arath [CaMV_virus_infection] : GST1.expr -> H2O2_local_accumulation ++  Arath [CaMV_virus_infection] : GST1.expr -> H2O2_systemic_accumulation ++  RSR -> H2O2 ++  RSR -> not_viral_systemic_signal //precede virus movement from infected leaf  Arath [CaMV_virus_infection] : PDF1_2.expr=present  Arath [CaMV_virus_infection] [ETR1,mut] : H2O2_accumulation=abolished //implicating ethylene signaling in the generation and transduction of the response_to_abscisic_acid_stimulus  Arath [CaMV_virus_infection] [EIN2,mut] : H2O2_accumulation=abolished //implicating ethylene signaling in the generation and transduction  Arath [CaMV_virus_infection] [NADPH,dmut_rbohDF] : H2O2_accumulation=abolished //implicating NADPH oxidase in the generation and transduction of the response of the response  NADPH_oxidase ~> ROS //generator of ROS  ethylene_madiated_signaling_pathway ~> ROS  Arath [CaMV_virus_infection] [ETR1,mut] : susceptibility_to_CaMV=reduced  Arath [CaMV_virus_infection] [EIN2,mut] : susceptibility_to_CaMV=reduced  Arath [CaMV_virus_infection] [EIN2,mut] : susceptibility_to_CaMV=reduced  Arath [CaMV_virus_infection] [NADPH,dmut_rbohDF] : susceptibility_to_CaMV=non_reduced //double mutant  Arath [CaMV_virus_infection] [NahG] : susceptibility_to_CaMV=identical //equal to wild type  !( salicylic_acid_madiated_signaling_pathway ~> susceptibility_to_CaMV)  ethylene_madiated_signaling_pathway ~> susceptibility_to_CaMV  ROS ~> susceptibility_to_CaMV |
| 12802336 | Pathogen-induced systemic plant signal triggers DNA rearrangements. | Kovalchuk I, Kovalchuk O, Kalck V, Boyko V, Filkowski J, Heinlein M, Hohn B. | Nature 2003 Jun 12;423(6941):760-2 | SPECIES: Nicotiana_tabacum  TMV = tobacco_mosaic_virus  ORMV = oilseed_rape_mosaic_tobamovirus  SRS = systemic_recombination_signal  TMV_virus_infection -> pathogen_induced_systemic_recombination_signal  ORMV_virus_infection -> pathogen_induced_systemic_recombination_signal  systemic_recombination_signal -> genomic_DNA_rearrangement  TMV_virus_infection -> genomic_DNA_rearrangement  ORMV_virus_infection -> genomic_DNA_rearrangement  (gene_duplication, chromosomal_duplication, unequal_crossing_over, deletion, insertion) (= genomic_DNA_rearrangements  Tobacco [TMV_virus_infection][N_resistance_gene_expr][lucyferase_transgene_regenerated] :_  homologous_recombination ++ @ infected_tissue //threefold increased  Tobacco [TMV_virus_infection][N_resistance_gene_expr][lucyferase_transgene_regenerated] :_  homologous_recombination ++ @ noninfected_tissue //threefold increased  TMV_virus.movement_speed < systemic_recombination_signal.movement_speed //homologues recombination travel faster than the virus can move  Tobacco [TMV_virus_infection][N_resistance_gene_expr][lucyferase_transgene_regenerated][signal_carrying_leaf_grafted] :_  homologous_recombination ++ @non_treated_plants //twofold increased  Tobacco [ORMV_virus_infection][N_resistance_gene_expr][lucyferase_transgene_regenerated][signal_carrying_leaf_grafted] :_  homologous_recombination ++ @non_treated_plants //twofold increased  Tobacco [ORMV_virus_infection][N_resistance_gene_expr][Su][su][signal_carrying_leaf_grafted]:_  homologous_recombination ++ @non_treated_plants //threefold and a half increased  Tobacco [SRS_induction] -> meiotic_recombination ++  Tobacco [SRS_induction] -> inherited_late_somatic_recombination ++ |
| 17311811 | Transgenerational changes in the genome stability and methylation in pathogen-infected plants: (virus-induced plant genome instability). | Boyko A, Kathiria P, Zemp FJ, Yao Y, Pogribny I, Kovalchuk I. | Nucleic Acids Res 2007;35(5):1714-25 | SPECIES: Nicotiana_tabacum  TMV = tobacco_mosaic_virus  SAR = systemic_acquired_resistance  R_genes = plant_resistant_genes  avr = pathogen_avirulence_gene  hypermethylation = addition_of_methyl_groups  hypomethylation = reduction_of_methyl_groups  LRR_loci = region_of_the_N-gene_of_resistance_to_TMV  RENT_loci = region_of_multiple_members_contain_moderately_repetitive_DNA  5.8S_r_RNA_loci = region_of_multiple_members  PI = progeny_of_infected_plants  PC = progeny_of_control_plants  Tobacco [TMV_virus_infection] : genomic_DNA_rearrangement ++  (gene_duplication, chromosomal_duplication, unequal_crossing_over, deletion, insertion) (= genomic_DNA_rearrangement  SAR (= defence_responce_to_virus  R_gene + avr -t> R_gene_avr //complex formation, R_gene^avr  R_gene_avr -| TMV_virus_infection  R_gene_avr -> defense_responce  R_gene_avr_interaction = incopatible_interaction  hypomethylation -> genom_instability  homologous_recombination -> genom_stability  hypermethylation -> genom_stability  methylation_changes -> rearrangement_in_resistence_gene_loci  Results  Tobacco [TMV_virus_infection_next_generation] : loci_containing_LRR_region=unstable //PI  Tobacco [TMV_virus_infection_next_generation] : rearragements_of_LRR_region.frequency=high //PI  Tobacco [TMV_virus_infection_next_generation] : RENT_coding_loci=stable //PI  Tobacco [TMV_virus_infection_next_generation] : 5.8_rRNA_coding_loci=stable //PI  Tobacco [TMV_virus_noninfection_next_generation] : RENT_coding_loci=stable //PC  Tobacco [TMV_virus_noninfection_next_generation] : 5.8_rRNA_coding_loci=stable //PC  TMV_virus_infection -> globally_hypermethylated_genom  Tobacco [TMV_virus_infection_next_generation] : loci_containing_LRR_region=hypometylation  Tobacco [TMV_virus_infection_next_generation] : actin_loci=hypermethylation //PI  Tobacco [TMV_virus_infection_next_generation] : methylation_status_of_actin_loci=increased //PI  Tobacco [TMV_virus_infection_next_generation] : methylation_status_of_RENT_coding_loci=identical //PI  Tobacco [TMV_virus_infection_next_generation] : methylation_status_of_5_8S_rRNA_coding_loci=identical //PI |
| 17555274 | Components of Arabidopsis defense- and ethylene-signaling pathways regulate susceptibility to Cauliflower mosaic virus by restricting long-distance movement. | Love AJ, Laval V, Geri C, Laird J, Tomos AD, Hooks MA, Milner JJ. | Mol Plant Microbe Interact 2007 Jun;20(6):659-70 | SPECIES: Arabidopsis  (salicylic_acid, jasmonic_acid, ethylene) (= signaling_molecules  (salicylic_acid, jasmonic_acid, ethylene) ~> defense_response_to_pathogen  CaMV = cauliflower_mosaic_virus  ROS = reactive_oxygen_species  salicylic_acid ~> pathogen_infection  Antimycin_A = inducer_of_resistance  (PR_1, Bgl_2, PR_5) = biomarkers_for_salicylic_acid_mediated_signaling_pathway  GST1 = biomarker_for_responce_to_ROS  PDF1_2 = biomarker_for_jasmonic_and_acid_ethylene_mediated_signaling_pathways  salicyl_hydroxamic_acid = inhibitor_of_alternative_oxidase  (EDS4, EDS8) (= jasmonic_acid_and_ethylene_mediated_signaling_pathways  EDS4 (= cross_talk_between_salicylic_acid_and_jasmonic_acid_and_ethylene_mediated_signaling_pathways  (ETR1, EIN2) (= ethylene_mediated_signaling_pathway  mut = mutation  Tobacco [TMV_virus_infection] : (salicylic_acid, cyanide, Antimycin_A) -> partial_resistans_to_TMV_  //Chivasa et al. 1997; Wong et al. 2002)  Arath [TVCV_virus_infection] : (salicylic_acid, cyanide, Antimycin_A) -> partial_resistans_to_TVCV_  //Chivasa et al. 1997; Wong et al. 2002)  Arath [CaMV_virus_infection] : (PR_1, PR_2, PR_5).expr ++  Arath [CaMV_virus_infection] : GST1.expr=present  Arath [CaMV_virus_infection] : PDF1_2.expr=present  Arath [SID2,mut] : salicylic_acid_biosynthetic_proces --  Arath [EDS5,mut] : salicylic_acid_biosynthetic_proces --  Arath [PAD4,mut] : salicylic_acid_biosynthetic_proces --  Arath [NahG] : salicylic_acid_biosynthetic_proces --  Results  Arath [SID2,mut][CaMV_virus_infection] : susceptibility_to_CaMV=non_enhanced  Arath [EDS5,mut][CaMV_virus_infection] : susceptibility_to_CaMV=non_enhanced  Arath [PAD4,mut][CaMV_virus_infection] : susceptibility_to_CaMV=non_enhanced  Arath [NahG][CaMV_virus_infection] : susceptibility_to_CaMV=non_enhanced  (CPR5, CPR1) -| salicylic_acid_biosynthetic_proces //Bowling et al. 1997; Clarke et al. 2001  Arath [CPR1,mut][CaMV_virus_infection] : salicylic_acid_biosynthetic_proces ++  Arath [CPR1,mut][CaMV_virus_infection] : resistance_to_CaMV=enhanced  Arath [CPR5,mut][CaMV_virus_infection] : salicylic_acid_biosynthetic_proces ++  Arath [CPR5,mut][CaMV_virus_infection] : resistance_to_CaMV=enhanced  Arath [CPR5, mut][EDS5,mut][CaMV_virus_infection] : resistance_to_CaMV=enhanced //resistance in cpr5 mutant may function partially independently of salicylic_acid  Arath [CPR1,mut][CaMV_virus_infection][salicyl_hydroxamic_acid_tritment] : susceptibility_to_CaMV=non_restored  Arath [EDS5,mut][CaMV_virus_infection][salicyl_hydroxamic_acid_tritment] : susceptibility_to_CaMV=partially_restored  Arath [CPR5,mut][EDS5,mut][CaMV_virus_infection][salicyl_hydroxamic_acid_tritment] : susceptibility_to_CaMV=partially_restored  Arath [ETR1,mut][EIN2,mut][EDS4,mut][EDS8,mut][CaMV_virus_infection] : susceptibility_to_CaMV=reduced  Arath [EDS4,mut][CaMV_virus_infection] : susceptibility_to_CaMV=reduced  Arath [EDS8,mut][CaMV_virus_infection] : susceptibility_to_CaMV=reduced  Arath [CPR1,mut][CaMV_GST_virus_infection] : susceptibility_to_CaMV=reduced //CaMV^GST  Arath [CPR5,mut][CaMV_GST_virus_infection] : susceptibility_to_CaMV=reduced //CaMV^GST  Arath [ETR1,mut][CaMV_GST_virus_infection] : susceptibility_to_CaMV=reduced //CaMV^GST  Arath [CPR1, mut][CaMV_GST_virus_infection] : systemic_movement_of_CaMV_GST=absent  Arath [CPR5, mut][CaMV_GST_virus_infection] : systemic_movement_of_CaMV_GST=absent  Arath [ETR1, mut][CaMV_GST_virus_infection] : systemic_movement_of_CaMV_GST=reduced |
| 16262714 | Coordinated activation of metabolic pathways for antioxidants and defence compounds by jasmonates and their roles in stress tolerance in Arabidopsis. | Sasaki-Sekimoto Y, Taki N, Obayashi T, Aono M, Matsumoto F, Sakurai N, Suzuki H, Hirai MY, Noji M, Saito K, Masuda T, Takamiya K, Shibata D, Ohta H. | Plant J 2005 Nov;44(4):653-68 | SPECIES: Arabidopsis  jasmonic_acid (= signaling_molecules  MeJA = methyl_jasmonate  (JA, MeJA) = jasmonates  JAs = jasmonates  (JA, MeJA) = ubiquitous_plant_signaling_compounds  JRGs = jasmonate_responsive_genes  ROS = reactive_oxygen_species  AsA = ascorbate_metabolic_pathway  GSH = glutathione_metabolic_pathway  AsA (= defence_responses_to_oxidative_stress  GSH (= defence_responses_to_oxidative_stress  indole_glucosinolate_biosyntesis = defence_compound_occurring_in_the_Brassicaceae_family  (AsA,GSH) = antioxidant_metabolic_pathways  ozon -> oxidative_stress  ozon -> antioxidant_methabolism  OPR3 = jasmonate_deficient_Arabidopsis_12_oxophytodienoate_reductase_3  AsA (= redox_compounds  AsA + ROS -t> AsA[oxidized] + ROS[reduced]  jasmonates ~> metabolic_pathways  jasmonats -> resistance_to_environmental_stresses  jasmonates -| virus_infection  jasmonic_acid_mediated_signaling_pathway ~> JRGs.expr  pathogen_infection -> jasmonic_acid_biosynthetic_process //endogenous accumulation  pathogen_infection -> JRGs.expr  jasmonic_acid_biosynthetic_proces -> JRGs.expr -- //McConn et al., 1997, Staswick et al., 1998, Thomma et al., 1998  componets_defect_in_jasmonic_acid_mediated_signaling_pathway -> JRGs.expr--_  //McConn et al., 1997; Staswick et al., 1998, Thomma et al., 1998  JRG.expr -> susceptibility_to_virus_infection ++  jasmonates -> AsA_methabolic_pathway_gene.expr //regulated by jasmonates at the transcriptional level  jasmonates -> GSH_methabolic_pathway_gene.expr //regulated by jasmonates at the transcriptional level  jasmonates -> indole_glucosinolate_biosynthetic_process_gene.expr  (ROS_elimination, antioxidants_accumulation) -| cell_death  virus_infection -> cell_death  //Results  (jasmonic_acid, tryptophan, serine, cysteine, glutathione_GSH,_  indole_glucosinolate, ascorbate_AsA).biosynthesis_pathways -> JRGs.expr //genes up-regulated by jasmonates  sulphur_assimilation_pathway -> JRGs.expr //genes up-regulated by jasmonates  AsA_recycling_pathway -> JRGs.expr //genes up-regulated by jasmonates  Arath [jasmonic_acid_tritment] : (GSH1, GSH2, DHAR, MDHAR, VTC1, VTC2).expr ++  jasmonic_acid -> (ascorbate, glutathione, cysteine).biosynthetic_process  jasmonic_acid -> dehydroascorbate_reductase_activity ++  Arath [ozon_treatment] : ROS ++ //oxidative burst  Arath [ozon_treatment] : (GSH2, MDHAR).expr=non_induced  Arath [ozon_treatment] : (GSH1, DHAR, VTC1, VTC2).expr=induced  Arath [OPR3,mut] [ozon_treatment] : (GSH1, DHAR, VTC1, VTC2).expr=abolished  Arath [OPR3,mut] [ozon_treatment] : sensitivity_to_oxidative_stress ++ |
| 12566575 | Quantitative nature of Arabidopsis responses during compatible and incompatible interactions with the bacterial pathogen Pseudomonas syringae. | Tao Y, Xie Z, Chen W, Glazebrook J, Chang HS, Han B, Zhu T, Zou G, Katagiri F. | Plant Cell 2003 Feb;15(2):317-30 | SPECIES: Arabidopsis  SUBJECT: Arabidopsis_responses_to_the_bacteria_pathogen  R_genes = plant_resistant_genes  avr = pathogen_avirulence_gene  Psp = bacterial_nonhost_pathogen_of_Arath_Pseudomonas_syringae_pv_phaseolicola_NPS3121  Pst = bacterial_virulent_strain_P_syringae_pv_tomato_DC3000  Psm = bacterial_virulent_strain_P_syringae_pv_maculicola_ES4326  mut = mutation  NahG = transgene_encoding_bacterial_salicylate_hydroxylase  ALG1 = specific_expression_marker_gene_for_RPS2_mediated_defence_responses  ELI3 = specific_expression_marker_gene_for_RPM1_mediated_defence_responses  Arath [avirulent_bacteria_infection][resistance_plant] : incompatible_host_pathogen_intereaction=present  Arath [virulent_bacteria_infection][susceptible_plant] : compatible_host_pathogen_interation=present  R_gene + avr -t> R_gene_avr //complex formation, R_gene^avr  R_gene_avr -> defense_response  R-gene_avr -> disease_resistance ++  (RPS2, RPM1) (= R_genes //in Arath  (avrRpt2, avrB) (= avr_genes //in Psp  avrRpt2 -> RPS2  avrB -> RPM1  //Results  Arath [Pst_bacterial_infection] : compatible_host_pathogen_interation=not_vigorous_response  Arath [Pst_bacterial_infection] [avrRpt2_expr] : incompatible_host_pathogen_interation=vigorous_response  Arath [Psp_bacterial_infection] : compatible_host_pathogen_interation=not_vigorous_response  Arath [Psp_bacterial_infection] [avrRpt2_expr] : incompatible_host_pathogen_interation=vigorous_response  Arath [Psp_bacterial_infection] [avrRpt2_expr] : RPM1_mediated_defence_responses=RPS2_mediated_defence_responses  Arath [NahG][Psp_bacterial_infection] [avrRpt2_expr] : RPS2_mediated_responses=suppressed  Arath [NahG][Psp_bacterial_infection] [avrRpt2_expr] : RPM1_mediated_responses=unsuppressed  Arath [NDR1,mut][Psp_bacterial_infection] [avrRpt2_expr]_  : RPS2_mediated_defence_responses=suppressed_circulating_follicle_stimulating_hormone_level  Arath [NDR1,mut][Psp_bacterial_infection] [avrRpt2_expr] : RPM1_mediated_defence_responses=unsuppressed  Arath [NahG][Psp_bacterial_infection] [avrB_expr] : RPS2_mediated_defence_responses=_  suppressed_circulating_follicle_stimulating_hormone_level  Arath [NahG][Psp_bacterial_infection] [avrB_expr] : RPM1_mediated_defence_responses=unsuppressed  Arath [NDR1,mut][Psp_bacterial_infection] [avrB_expr] : RPS2_mediated_defence_responses=suppressed  Arath [NDR1,mut][Psp_bacterial_infection] [avrB_expr] : RPM1_mediated_defence_responses=unsuppressed  Arath [NahG][Psp_bacterial_infection] [avrB_expr] : RPS2_mediated_defence_responses=suppressed  Arath [NahG][Psp_bacterial_infection] [avrB_expr] : RPM1_mediated_defence_responses=unsuppressed  Arath [NDR1,mut][Psp_bacterial_infection] [avrB_expr] : RPS2_mediated_defence_responses=suppressed  Arath [NDR1,mut][Psp_bacterial_infection] [avrB_expr] : RPM1_mediated_defence_responses=unsuppressed |
| 17360504 | Arabidopsis SNI1 and RAD51D regulate both gene transcription and DNA recombination during the defense response. | Durrant WE, Wang S, Dong X. | Proc Natl Acad Sci U S A 2007 Mar 6;104(10):4223-7 | SPECIES: Arabidopsis  mut = mutation  PR_genes = pathogenesis_related_genes  SAR = systemic_acquired_resistance  PR_proteins = pathogenesis_related_proteins  salicylic_acid (= signaling_molecules  salicylic_acid ~> defense_response  NPR1= nonexpresser_of_PR_genes_1 //transcriptional coactivator  SNI1 = suppressor_of_NPR1_inducible_1 //transcriptional repressor  INA = 2_6_dichloroisonicotinic_acid  INA = salicylic_acid_analogue  BTH = salicylic_acid_analogue  BTH = benzothiadiazole_S_methyl_ester  RAD51D = suppressor_of_sni1_1 //genetic screen for suppressors  salicylic_acid -> PR_genes.expr  salicylic_acid -> NPR1.expr  (Bgl2,PR_1) (= biomarkers_for_salicylic_acid_mediated_signaling_pathway  Psm = bacterial_virulent_strain_P_syringae_pv_maculicola_ES4326  MMC = DNA_cross_linking_agent_mitomycin_C  bleomycin -> doble_strand_breaks  Arath [BTH_tritment] : somatic_homologous_recombination ++  Arath [INA_tritment] : somatic_homologous_recombination ++  Arath [SNI1,mut] [NPR1,mut] : PR_genes.expr=induced //salicylic acid required to fully induce PR-genes and SAR  Arath [SNI1,mut] [NPR1,mut] : SAR=restored //salicylic acid required to fully induce PR-gene and SAR  Arath [SNI1,mut] [NPR1,mut] : leaves=narrow  Arath [SNI1,mut] : growth --  Arath [NPR1,mut] : PR_genes.expr=abolished  Arath [NPR1,mut] : resistance_to_virus=abolished  Results  Arath [RAD51D,mut][SNI1,mut] : morphology=restored  Arath [RAD51D,mut][SNI1,mut] : enhanced_PR_genes.expr=abolished  Arath [RAD51D,mut][SNI1,mut] : DNA_recombination=abolished  Arath [RAD51D,mut][SNI1,mut][NPR1,mut] : morphology=restored  Arath [RAD51D,mut][SNI1,mut][NPR1,mut] : enhanced_PR_genes.expr=abolished  Arath [RAD51D,mut][SNI1,mut][NPR1,mut] : DNA_recombination=abolished  Arath [BGL2_GUS][RAD51D,mut] : BGL2_GUS.expr=abolished //BGL^GUS  Arath [BGL2_GUS_expr][RAD51D,mut][SNI1,mut][NPR1,mut] : BGL2_GUS.expr=abolished  Arath [BGL2_GUS_expr][RAD51D,mut][SNI1,mut][BTH_tritment] : BGL2_GUS_expr=induced  Arath [BGL2_GUS_expr][RAD51D,mut][SNI1,mut][NPR1,mut][BTH_tritment] : BGL2_GUS.expr=none //resembling npr1 mutant  Arath [PR_1_expr][INA_tritment][SNI1,mut] : PR_1.expr=induced //at 10-fold lower concentration of INA than wild type  Arath [PR_1_expr][INA_tritment][SNI1,mut][NPR1,mut] : PR_1.expr=induced //at 10-fold lower concentration of INA than WT  Arath [PR_1_expr][INA_tritment][SNI1,mut][RAD51D,mut] : PR_1.expr=induced //wild type pattern of induction was restored  Arath [PR_1_expr][INA_tritment][SNI1,mut][NPR1,mut][RAD51D,mut] : PR_1.expr=none //PR1 induction pattern reverted to that of npr1 mutant  Arath [NPR1,mut] : susceptibility_to_Psm ++  Arath [RAD51D,mut][SNI1,mut][NPR1,mut][Psm_bacterial_infection] : susceptibility_to_Psm ++  Arath [RAD51D,mut][Psm_bacterial_infection] : susceptibility_to_Psm ++  Arath [SNI1,mut][Psm_bacterial_infection] : susceptibility_to_Psm ++  Arath [SNI1,mut][NPR1,mut][Psm_bacterial_infection] : susceptibility_to_Psm ++  Arath [RAD51D,mut][MMC_tritment] : DNA_cross_linking=repair  Arath [RAD51D,mut][bleomycin_tritment] : doble_strand_breaks=repair  (SNI1, RAD51D) ~> homologous_recombination_repaired_pathway  (SNI1, RAD51D) ~> defence_gene_transcription  Arath [SNI1,mut][GUS_expr] : recombination_level=elevated  Arath [SNI1,mut][RAD51D,mut][GUS_expr] : recombination_level=non_elevated |
| 17615233 | Infection and coaccumulation of tobacco mosaic virus proteins alter microRNA levels, correlating with symptom and plant development. | Bazzini AA, Hopp HE, Beachy RN, Asurmendi S. | Proc Natl Acad Sci U S A 2007 Jul 17;104(29):12157-62 | SPECIES: Nicotiana_tabacum  TMV = tobacco_mosaic_virus  ToMV = tomato_mosaic_virus  TEV = tobacco_etch_virus  PVY = potato_virus_Y  PVX = potato_virus_X  (TMV, ToMV) (= tobamovirus_family  PTGS = posttranscriptional_gene_silencing  MP = tobacco_mosaic_virus_movement_protein  CP = tobacco_mosaic_virus_coat_protein  pm = point_mutation  (TEV, PVY) (= potyvirus_family  PVX (= potexvirus_family  small_RNAs ~> plant_growth_and_development  small_RNAs ~> host_pathogen_interaction  (siRNAs, miRNAs) (= small_RNAs  siRNAs (= PTGS_system  PTGS -| virus_infection  PTGS_system_suppresors (= viral_proteins  PTGS_system_suppresors -| PTGS_pathway  PTGS_system_suppresors -> virus_replication ++  miRNAs ~> gene_expression // in plants and animals  miRNAs ~> (plant_development, signal_transduction, protein_degradation, response_to_environmental_stress, pathogen_infection)  (TMV, ToMV) -> miRNA_accumulation ++  (TEV, PVY) -> miRNA_accumulation //moderate changes  Tobacco [TMV_virus_MP_expr] : resistance_to_TMV_virus_infection --  Tobacco [TMV_virus_infection]: miRNAs_accumulation ++  TEV.PTGS_supressor_activity=strong  PVY.PTGS_supressor_activity=strong  TMV.PTGS_supressor_activity=week  PVX.PTGS_supressor_activity=week  ToMV.PTGS_supressor_activity=inactive  TMV -> (virus_MP, virus_CP).expr  Tobacco [TMV_virus_CP_expr] : resistance_to_TMV_virus_infection ++  Tobacco [TMV_virus_MP_expr] : cell_to_cell_TMV_virus_movement ++  //Results  Tobacco [TMV_virus_CP_pmT42W_expr] : protein_agregation ++  Tobacco [TMV_virus_CP_pmT42W_expr] : CP_madiated_resistance ++  !(TMV_virus_CP.expr -| posttranscriptional_gene_silencing)  !(TMV_virus_MP.expr -| posttranscriptional_gene_silencing)  (TMV, ToMV) -> miRNA_accumulation ++  (TEV, PVY) -> miRNA_accumulation //moderate changes  Tobacco [TMV_virus_MP_expr] : resistance_to_TMV_virus_infection --  Tobacco [TMV_virus_CP_pmT42W_expr]: resistance_to_TMV_virus_infection --  Tobacco [TMV_virus_MP_expr] [TMV_virus_CP_pmT42W_expr]: resistance_to_TMV_virus_infection ++  Tobacco [TMV_virus_MP_expr] [TMV_virus_CP_pmT42W_expr]: miRNAs_accumulation ++  Tobacco [TMV_virus_infection]: miRNAs_accumulation ++ |
| 16435264 | Fine-Tuning Plant Defence Signalling: Salicylate versus Jasmonate. | Beckers GJ, Spoel SH. | Plant Biol (Stuttg) 2006 Jan;8(1):1-10 | SAR = systemic_acquired_resistance  PR_genes = pathogenesis_related_genes  TGA = transcription_factor  as_1 = activator_sequence_1  COI1_ARATH = coronatine_insensitive_protein_1  COI1 (= positive_regulator_of_jasmonic_acid_mediated_signaling_pathway  LOX2_arath = lipoxygenase_chloroplast_precursor_gene  DAD1 = gene_encodes_jasmonic_acid_inducible_chloroplast_localized_phospholipase_A_1  AOS = allele_oxide_synthase  AOC = allele_oxide_cyklas  OPR3 = OPDA_reduktase_3  OPDA = 12_oxo_phytodienoic_acid  SKP1A_ARATH = SKP1_like_protein_A  cullin1 = SCF_dependent_proteasomal_ubiquitin_dependent_protein_catabolic_process  SCF = SKP1_cullin1_F_box_protein_complex  (biotrophic_pathogens, necrotrophic_pathogens) (= microbial_pathogens  biotrophic_pathogens -> defence_response_dependent_on_salicylic_acid  necrotrophic_pathogens -> defence_response_dependent_on_jasmonic_acid  (salicylic_acid, jasmonic_acid, ethylene) (= signaling_molecules  (salicylic_acid, jasmonic_acid, ethylene) ~> defence_response  NPR1= nonexpresser_of_PR_genes_1 //transcriptional coactivator  salicylic_acid -> PR_genes.expr  salicylic_acid -> NPR1  salicylic_acid ~> SAR_signaling_transduction  (JAR1, COI1) (= jasmonic_acid_mediated_signaling_pathway //Staswick et.al., 1992; Feys et al., 1994  COI1 -> jasmonic_acid_mediated_signaling_pathway  jasmonic_acid -> COI1  COI1 + SKP1 + cullin1 -t> COI1_SKP1_cullin1 //COI1^SKP1^cullin1  Arath [NPR1,mut] : npr1-| salicylic_acid_mediated_defence_response  Arath [NPR1,mut][virus_infection] : salicylic_acid_biosynthetic_process ++  Arath [virus_infection] : NPR1.expr++  Arath [salicylic_acid_treatment] : NPR1.expr++  //Cannot add yet in MineMap:// Arath [NPR1++] [virus_infection] : pathogenesis_related_genes.expr++  salicylic_acid -> NPR1-t> NPR1[@cytosol]  NPR1[@cytosol] -| jasmonic_acid_mediated_signaling_pathway  salicylic_acid -> NPR1 -t> NPR1 [@nucleus]  NPR1 [@nucleus] + TGA -t> NPR1_TGA //NPR1^TGA  NPR1_TGA -s> PR_genes  TGA + as_1 -t> TGA_as_1 //TGA^sa_1  TGA_as_1 -> PR_genes.expr  jasmonic_acid ->(DAD1,LOX2,AOS,AOC,OPR3).expr_  //Bell and Mullet, 1993; Kubigsteltig et al., 1999; Mussig et al., 2000; Ishiguro et al., 2001;Spoel et al., 2003; Stenzel et al., 2003  jasmonic_acid -| root_growth  Arath [salicylic_acid_treatment] : VSP.expr=suppressed  salicylic_acid_mediated_signaling_pathway -| jasmonic_acid_mediated_signaling_pathway  Arath [NPR1,mut] : npr1-| salicylic_acid_mediated_signaling_pathway |
| 15283665 | Systemic acquired resistance. | Durrant WE, Dong X. | Annu Rev Phytopathol 2004;42:185-209 | SPECIES: Arabidopsis  R_genes = resistance_genes  SAR = systemic_acquired_resistance  PR_genes = pathogenesis_related_genes  NPR1 = nonexpresser_of_PR_genes_1 //transcriptional coactivator  salicylic_acid (= signaling_molecules  salicylic_acid ~> SAR  DIR1 = defective_in_induced_resistance1  DIR1 = LTP2-like_resistance_signaling_protein  LTPs = lipid_transfer_proteins  LPT2 (= LTPs  DIR1 (= SAR  ROS = reactive_oxygen_species  EDS1 = essential_component_of_R_gene_mediated_disease_resistance_in_Arath  PAD4 = phytoalexin_deficient4  PAD4 = lipase_like_gene_important_for_salicylic_acid_mediated_signaling_pathway  SFD1 = suppressor_of_fatty_acid_desaturase_deficiency_1  SFD1 (= glicerolipid_methabolism  SFD1 -> SAR  ICS1 = isochorismate_synthase_1  ICS1.expr -> salicylic_acid ++  ICS1 ~> salicylic_acid_biosynthesis  ICS1.expr -> pathogen_resistance  IPL1 = isochorismate_pyruvate_lyase_1  IPL1.expr -> salicylic_acid ++  IPL1.expr -> pathogen_resistance  SID2/EDS16 = encode_a_putative_chloroplast_localized_ICS1  SID1/EDS5 (= salicylic_acid_mediated_signaling_pathway //Nawrath et al., 2002  EDS5 (= disease_resistance  EDS5 (= member_of_the_META_transporter_family  (RPS4, EDS1 ,PAD4) ~> salicylic_acid_biosynthesis  (RPS4, EDS1 ,PAD4) (= R_genes  (NIMIN1, MININ2, NIMIN3) = NIM_interactors  NIMIN1 + NPR1 -t> NIMIN1_NPR1 //interaction with C terminus of NPR1  NIMIN2 + NPR1 -t> NIMIN2_NPR1 //interaction with C terminus of NPR1  NIMIN3 + NPR1 -t> NIMIN3_NPR1 //interaction with N terminus of NPR1  (TGA2, TGA3, TGA5, TGA6, TGA7) (= transcription_factors_interacted_with_NPR1  (TGA1, TAG4) (= transcription_factors_weakly_or_not_interacted_with_NPR1  (TGA2, TGA5, TGA6) ~> SAR  salicylic_acid ~> TGA1_redox_status  salicylic_acid ~> TGA4_redox_status  SNI1 -| PR_gene.expr  SNI1 -| SAR  (SSN1, SSN2, SSN3) ~> PR_gene.expr //basal and SA-inducible  AtWhy1 (= Whirly_family_of_transcription_factors  !(NPR1 -> AtWhy1)  DTH9 ~> SAR  !(DTH9 ~> NPR1)  salicylic_acid ~> DTH9 -> PR_gene.expr  salicylic_acid ~> AtWH1-> PR_gene.expr  Arath [DIR1,mut] : phloem_sap=deficient //in the mobil signal for SAR |
| 18316638 | Cross talk in defense signaling. | Koornneef A, Pieterse CM. | Plant Physiol 2008 Mar;146(3):839-44 | SPECIES: Arabidopsis  VSP2 = vegetative_storage_protein2  LOX2 = lipoxygenase2  PDF1_2 = plant_defensin1_2  MYC2 = transcription_factor  ERF1 = ethylene_response_factor1  GRX480 = glutaredoxin_interacted_with_TGAs  EDS1 = enhanced_disease_susceptibility1  PAD4 = phytoalexin_deficient4  MKS1 = MAP_kinase_4_substrate1  abscisic_acid (= plant_hormones  biotrophic_pathogens -> salicylic_acid_mediated_defence_response  necrotrophic_pathogens -> jasmonic_acid_mediated_defence_response  salicylic_acid -> PR_1.epxr  MeJA -> PDF1_2.expr  salicylic_acid -| PDF1_2.expr //on the jasmonic acid mediated defence response  jasmonic_acid -> MYC2_dependent_gene.expr //in response to wounding  abscisic_acid -> MYC2_dependent_gene.expr //in response to wounding  jasmonic_acid_ethylene_combined_action -> ERF1_dependent_gene.expr //in response to pathogen attack  salicylic_acid ~> SAR  salicylic_acid -> NPR1  NPR1 (= cross_talk_between_  salicylic_acid_and_jasmonic_acid_mediated_signaling_pathways  (WRKY70, WRKY11, WRKY17, WRKY62, WRKY25, WRKY33) (= transcription_factors  Arath [WRKY70 ++] : PR_genes.expr ++, PDF1_2.expr=suppressed //Li et al., 2004  (WRKY70, WRKY11, WRKY17, WRKY62) (= cross_talk_between_salicylic_acid_and_jasmonic_acid_mediated_signaling_pathways  salicylic_acid -> NPR1  NPR1 -> GRX480  salicylic_acid -> NPR1 -> GRX480  GRX480 + TGA -t> GRX480_TGA //GRX480^TGA  GRX480_TGA -| jasmonic_acid_induced_genes.expr //GRX480^TGA  Arath [GRX480++] : PDF1_2.expr=abolished, LOX2.expr=induced, VSP2.expr=induced  jasmonic_acid -> (LOX2, VSP2).expr  MPK4 = MAP_kinase4  MPK4 -| salicylic_acid_mediated_signaling_pathway  MPK4 -> jasmonic_acid_mediated_signaling_pathway  Arath [MPK4,mut] : salicylic_acid_biosynthesis++  Arath [MPK4,mut] : PR_genes.expr ++  Arath [MPK4,mut] : jasmonic_acid_induced_genes.expr  Arath [MPK4,mut] : susceptibility_to_A_brassicicola=enhanced  (EDS1, PAD4) (= downstream_effectors_of_MPK4  (EDS1, PAD4) -> salicylic_acid_mediated_signaling_pathway //Broderrsen et al., 2006  (EDS1, PAD4) -| jasmonic_acid_mediated_signaling_pathway //Broderrsen et al., 2006  MKS1 [P] -| salicylic_acid_mediated_signaling_pathway  MKS1 + WRKY25 -t> MKS1_WRKY25 // MKS1^WRKY25, Anderson et al., 2005  MKS1 + WRKY33 -t> MKS1_WRKY33 // MKS1^WRKY33, Anderson et al., 2005 |
| 16759898 | Crosstalk between abiotic and biotic stress responses: a current view from the points of convergence in the stress signaling networks. | Fujita M, Fujita Y, Noutoshi Y, Takahashi F, Narusaka Y, Yamaguchi-Shinozaki K, Shinozaki K. | Curr Opin Plant Biol 2006 Aug;9(4):436-42 | SPECIES: Arabidopsis  ROS = reactive_oxygen_species  del = deletion  MYC2 = transcription_factor  R2R3MYB2 = transcription_factor_encoded_by_BOS1  BOS1 = Botrytis_susceptible1  NAC = transcription_factor_encoded_by_RD26  ATAF2 = member_of_the_plant_specific_NAC_domain_transcription_factor_family  MAPKKK1 = mitogen_activated_protein_kinase_kinase_kinase1  NDPK2 = nucleoside_diphosphate_kinase2  (rbohD, rbohF) = NADPH_dependent_respiratory_burst_oxidase_homolog_genes  Zat12 = C2H2_type_zinc_finger_transcription_factor12  APX1 = ascorbate_peroxidase1  HSF21 = redox_sensitive_transcription_heat_shock_factor21  (RPW8_1, RPW8_2) = disease_resistance_R_genes  SLH1 = sensitive_to_low_humidity  (MLA1, MLA6) = barlay_mildew_MLA_resistance_proteins  (salicylic_acid, jasmonic_acid, ethylene, abscisic_acid)(= phytohormones  response_to_abiotic_stress -> ROS.generation  response_to_biotic_stress -> ROS.generetion  response_to_abiotic_stress -> phytohormones.biosynthesis  response_to_biotic_stress -> phytohormones.biosynthesis  response_to_abiotic_stress -> abscisic_acid_response //dominant process for abscisic_acid, Anderson et al., 2004  MYC2 (= response_to_drought  Arath [MYC2++] [MYB2++] : abscisic_acid_sensitivity=high, osmotic_stress_response=enhanced  BOS1 (= response_to_abiotic_stress //via ROS  BOS1 (= response_to_biotic_stress //via ROS  (jasmonic_acid, hydrogen_peroxide, pathogen, drought_environment ,salinity, abscisic_acid) -> RD26.expr  ATAF2 -| pathogenesis_related_protein  Arath [ATAF2++] [acetic_acid++] : pathogenesis_related_protein=repressed  Arath [ATAF2++] [jasmonic_acid++] : pathogenesis_related_protein=repressed  Arath [ATAF2++] [high_salinity++] : pathogenesis_related_protein=repressed  Arath [ATAF2++] [high_salinity++] : pathogenesis_related_protein=repressed  Arath [ATAF2++] [wounding++] : pathogenesis_related_protein=repressed  response_to_abiotic_stress -> (MPK4, MPK6, MPK3)  response_to_oxidative_stress -> MPK6  response_to_ROS -> (MPK3, MPK4) //affected by OXI1  MAPKKK1 -> (MPK3, MPK6) //via H2O2  H202 -> NDPK2.expr  NDPK2 + MPK3 -t> NDPK2_MPK3 //specific interaction  NDPK2 + MPK6 -t> NDPK2_MPK6 //specific interaction  Arath [NDPK2++] : cold_tolerance=enhanced  Arath [NDPK2++] : salt_tolerance=enhanced  Arath [NDPK2++] : oxidative_stress=enhanced  (rbohD, rbohF) (= ROS.generation  Zat12 ~> ROS_scavenging_mechanism  ROS_scavenging_mechanism (= response_to_abiotic_stress  Arath [Zat12, del] : APX1.expr=suppressed  Arath [Zat12++] : oxidative_stress_responsive_genes.expr=upregulated, light_stress_responsive_genes.expr=upregulated  Arath [Zat12++] : hight_light_tolerance=enhanced  Arath [Zat12++] : freezing_tolerance=enhanced  Arath [Zat12++] : oxidative_stress=enhanced  Arath [RPW8++] [high_temperature_treatment++] : cell_death_phenotype=suppressed  Arath [RPW8++] [humidity_treatment++] : cell_death_phenotype=suppressed  Arath [SLH1,mut] [high_temperature_treatment] : cell_death_phenotype=suppressed  Arath [SLH1,mut] [humidity_treatment] : cell_death_phenotype=suppressed  Arath [SLH1,mut] : salicylic_acid_biosynthesis=induced //Noutoshi et al., 2005  Arath [SSI4,mut] [humidity_treatment]: MPK3_activation=inhibited  Arath [SSI4,mut] [humidity_treatment]: MPK6_activation=inhibited  Arath [SSI4,mut] [humidity_treatment] : H2O2_biosynthesis=inhibited  Arath [SSI4,mut] : salicylic_acid_biosynthesis=induced //Shirano et al., 2002 |
| 18273012 | Dual control of nuclear EIN3 by bifurcate MAPK cascades in C2H4 signalling. | Yoo SD, Cho YH, Tena G, Xiong Y, Sheen J. | Nature 2008 Feb 14;451(7180):789-95 | Arath  Ethylene ~> _  (germination, root_develoment, shoot_development, flower_development, response_to_stress, response_to_glucose_stimulus,_  fruit_ripening, senescence)  CTR1 -| ethylene_mediated_signaling_pathway  CTR1 = serine_threonine_protein_kinase  CTR1 = homologous_to_the_RAF_like_MAPKKK_kinase  CTR1 = constitutive_triple_response1  CTR1 + ETR1 -t> CTR1_ETR1 //interaction  EIN2 = ethylene_insensitive2_protein  EIN3 = ethylene_insensitive3_protein  EIN3 = nuclear_transcription_factor  ETR1 = ethylene_response1_receptor  ELI1 = nuclear_transcription_factor  ELI1 = EIN3_like1_protein  EIN3 -> ethylene_mediated_signaling_pathway  ELI1 -> ethylene_mediated_signaling_pathway  EIN3 + EBF1 -t> EIN3_EBF1 //interaction  EIN3 + EBF2 -t> EIN3_EBF1 //interaction  (EBF1, EBF2) = F_box_proteins  (hormone, stress, microbial_elicitor, developmental_process) -> (MPK3, MPK6)  (ACS2, ACS6) = ethylene_biosynthetic_enzymes  response_to_abiotic_stress -> MPK6  response_to_biotic_stress -> MPK6  MPK6 -> (P-> ACS2)  MPK6 -> (P-> ACS6)  ACS2[P] -> ethylene_synthesis  ACS6[P] -> ethylene_synthesis  ethylene -| (ETR1, ERS1)  (ETR1/ERS1) -> CTR1  (MKK9_MPK3, MKK9_MPK6) ~> nuclear_EIN3_stability  MKK9_MPK3 -> (P->EIN3)  EIN3[P,T174] -> nuclear_EIN3_stability  MKK9_MPK6 -> (P->EIN3)  EIN3[P,T174] -> nuclear_EIN3_stability  CTR1 -> (P->EIN3)  EIN3[P,T592] -| nuclear_EIN3_stability  MAPK_cascades (= ethylene_mediated_signaling_pathway  CTR1 -| MKK9_MPK3 //MKK9_MPK3 cascade  CTR1 -| MKK9_MPK6 //MKK9_MPK6 cascade  (MPK3, MPK6)(= ethylene_mediated_signaling_pathway  (MKK4, MKK5, MKK7, MKK9)-> (MPK3, MPK6) //MKK4, MKK5, MKK7, MKK9 constitutively active  Arath [MKK7++][MKK9++] : MPK3.expr=activated, MPK6.expr=activated  Arath [MKK7++][MKK9++][CTR1--] : MPK3.expr=activated, MPK6.expr=activated //no endogenous CTR1 activity  Arath [MKK4++][MKK5++] : MPK3.expr=not_activeted, MPK6.expr=not_activated  Arath [MKK4++][MKK5++][CTR1--] : MPK3.expr=not_activeted, MPK6.expr=not_activated //no endogenous CTR1 activity |
| 18539774 | Kinetics of salicylate-mediated suppression of jasmonate signaling reveal a role for redox modulation. | Koornneef A, Leon-Reyes A, Ritsema T, Verhage A, Den Otter FC, Van Loon LC, Pieterse CM. | Plant Physiol 2008 Jul;147(3):1358-68 | Species: Arath  coronatine = jasmonate_analog  coronatine = phytotocsin  coronatine -| defences_dependent_of_salicylic_acid  WRKY70 = transcription_factor  WRKY70 -> salicylic_acid_responsive_genes  WRKY70 -| jasmonic_acid_indusible_genes  salicylic_acid -| (PDF1_2, LOX2, VSP2).expr //salicylic acid response either by pathogen infection or by exogenous application  Arath [NPR1,mut] [salicylic_acid_treatment] : (PDF1_2, LOX2, VSP2).expr=induced  GRX480 -s| PDF1_2 //Ndamukong et al., 2007  glutatione = redox_potential_marker  BSO = glutatione_biosyntesis_inhibitor  BSO = L_buthionine_sulfoximine  //Results  (A_brassicicola, B_cinerea) (= necrotrophic_funguses  (F_occidentalis, P_rapae)(= herbivorous_insects  (A_brassicicola, B_cinerea, F_occidentalis, P_rapae)(= jasmonic_acid_inducers  H_parasitica (= salicylic_acid_inducing_biotrophic_pathogens  Arath [salicylic_acid_treatment] : PR_1.expr=induced  Arath [necrotrophic_fungus] : PDF1_2.expr=induced  Arath [necrotrophic_fungus][salicylic_acid_treatment] : PR_1.expr=induced  Arath [necrotrophic_fungus][salicylic_acid_treatment] : PDF1_2.expr=suppressed  Arath [herbivorous_insect] : PDF1_2.expr=induced  Arath [herbivorous_insect] : VSP2.expr=induced  Arath [herbivorous_insect][salicylic_acid_treatment] : PR_1.expr=induced  Arath [herbivorous_insect][salicylic_acid_treatment] : PDF1_2.expr=suppressed  Arath [herbivorous_insect][salicylic_acid_treatment] : VSP2.expr=suppressed  Arath [MeJA_treatment][salicylic_acid_treatment] : PR_1.expr=induced  Arath [MeJA_treatment][salicylic_acid_treatment] : PDF1_2.expr=suppressed  Arath [H_parasitica] : PR_1.expr=induced  Arath [MeJA_treatment] : PDF1_2.expr=induced  Arath [MeJA_treatment] : VSP2.expr=induced  Arath [H_parasitica][MeJA_treatment] : PR_1.expr=induced  Arath [H_parasitica][MeJA_treatment] : PDF1_2.expr=suppressed  Arath [H_parasitica][MeJA_treatment] : VSP2.expr=suppressed  Arath [H_parasitica] : PR_1.expr=induced  Arath [P_rapae] : VSP_2.expr=induced  Arath [H_parasitica][P_rapae] : PR_1.expr=induced  Arath [H_parasitica][P_rapae] : PDF1_2.expr=suppressed  Arath [salicylic_acid_treatment][MeJA_treatment] : antagonistic_effect_of_salicylic_acid_on_  _jasmonic_acid_resposive_gene=(vigorous, sensitive, rapid, long_lasting, transient).response  Arath [CPR1,mut][MeJA_treatment] : PR_1.expr=induced  Arath [CPR1,mut][MeJA_treatment] : PDF1_2.expr=reduced  Arath [salicylic_acid_treatment][MeJA_treatment][SBO_treatment] : PDF1_2.expr=non_suppressed |
| 15053755 | Systemic plant signal triggers genome instability. | Filkowski J, Yeoman A, Kovalchuk O, Kovalchuk I. | Plant J 2004 Apr;38(1):1-11 | SPECIES: Nicotiana_tabacum  SRS = systemic_recombination_signal  NahG = naphtalene_degrading_salicylate_1_hydroxylase  homologous_recombination ~> gene_rearranged_at_DNA_level  (UVC, rose_Bengal)(= DNA_damaging_agents  UVC -> ROS //generate ROS  RB = rose_Bengal  NAC = N_acetyl_L_cysteine  NAC = radical_scavenging_compound  local_treatments -> systemic_recombination_signal  grafting -> systemic_signal  grafting = non_treated_leaves_from_treated_plants_onto_healty_plants  local_tissue = treated_tissue  systemic_tissue = non_treated_tissue  //Results  Tobacco [lucyferase_trangene_regenerated][UVC_treatment] : homologous_recombination ++  Tobacco [lucyferase_trangene_regenerated][RB_treatment] : homologous_recombination ++  Tobacco [lucyferase_trangene_regenerated][UVC_treated_leaf][RB_treated_leaf] : homologous_recombination ++ @in_treated_leaves  Tobacco [lucyferase_trangene_regenerated][UVC_treated_leaf][RB_treated_leaf] : homologous_recombination ++ @non_treated_leaves  Tobacco [UVC_treated_leaf][lucyferase_transgene_regenerated][signal_carrying_leaf_grafted] :_  homologous_recombination ++ @non_treated_plants  Tobacco [RB_treated_leaf] [lucyferase_transgene_regenerated][signal_carrying_leaf_grafted] :_  homologous_recombination ++ @non_treated_plants  Tobacco [NahG][UVC_treated_leaf][lucyferase_transgene_regenerated][signal_carrying_leaf_grafted] :_  homologous_recombination ++ @non_treated_plants  Tobacco [NAC_treatment][NahG][UVC_treated_leaves][lucyferase_transgene_regenerated][signal_carrying_leaf_grafted] :_  homologous_recombination -- @non_treated_plants  Tobacco [NAC_treatment][NahG][RB_treated_leaves][lucyferase_transgene_regenerated][signal_carrying_leaf_grafted] :_  homologous_recombination -- @non_treated_plants  Tobacco [NAC_treatment][UVC_treated_leaves][lucyferase_transgene_regenerated][signal_carrying_leaf_grafted] :_  homologous_recombination -- @non_treated_plants  Tobacco [NAC_treatment][RB_treated_leaves][lucyferase_transgene_regenerated][signal_carrying_leaf_grafted] :_  homologous_recombination -- @non_treated_plants |
| 16760490 | Reactive oxygen species signaling in response to pathogens. | Torres MA, Jones JD, Dangl JL. | Plant Physiol 2006 Jun;141(2):373-8 | ROS = reactive_oxygen_species  NADPH_oxidase = respiratory_burst_oxidase  RBO = respiratory_burst_oxidase  MAMPs = microbe_associated_molecular_patterns  SAR = systemic_acquired_resistance  defence_response -> oxidative_burst  ETR1 = ethylene_receptor  DPI = diphenylene_iodonium  DPI -| plasma_membrane_NADPH_oxidase  (cyanide, azide) -| cell_wall_peroxidases  Rboh_family_genes = NADPH_dependent_respiratory_burst_oxidase_homolog_genes  NADPH_oxidase ~> induction_by_symbiont_of_defense_related_host_reactive_oxygen_species_production  response_to_pathogen -> NADPH_oxidase -> ROS_generation  (salicylic_acid, nitric_oxide)(= regulation_of_respiratory_burst  (ROS, salicylic_acid) ~> SAR  ethylene_madieted_signaling_pathway ~> ROS  ETR1 ~> cross_talk_between_ethylene_and_hydrogen_peroxidase  Arath [avr_bacteria][rbohDF] : hypersensitive_response -- |
| 17215350 | Arabidopsis systemic immunity uses conserved defense signaling pathways and is mediated by jasmonates. | Truman W, Bennett MH, Kubigsteltig I, Turnbull C, Grant M. | Proc Natl Acad Sci U S A 2007 Jan 16;104(3):1075-80 | Species = Arath  compatible_interaction -> avr_gene  avr_gene -> hypersensitive_response  hypersensitive_response -> pathogen_localization -> SAR  local_tissue = treated_tissue  systemic_tissue = non_treated_tissue  incompatible_interaction -> R_gene_avr_gene //R_gene^avr_gene  R_gene_avr_gene -> systemic_pathogen_spread  systemic_pathogen_spread -> symptoms_development  mut = mutation  avrB = avirulentof_Pseudomonas_syringae_pv_tomato_DC3000_immunnizing_challenge  hrpA = mutant_of_Pseudomonas_syringae_pv_tomato_DC3000_immunizing_challenge  SAR = systemic_acquired_resistance  salisylic_acid (= (local_response, systemic_response)  salicylic_acid ~> SAR //SAR does not require long distance translocation of salicylic acid  jasmonates -> SAR //rapid induction  jasmonates -> systemic_response  Arath [avrB] : jasmonic_acid ++ //rapid accumulation in phloem  PAMPs = pathogen_associated_molecular_patterns  PIGs = PAMPs_induced_genes  A70 (= PIGs  Arath [RPM1,mut][ATRAR1,mut][systemic_tissues][avrB]: A70.expr=not_induced  Arath [RPM1,mut][ATRAR1,mut][systemic_tissues][Pto_DC3000]: A70.expr=not_induced  Arath [NahG][systemic_tissues][avrB] : A70.expr=induced //induction near wild type  Arath [systemic_tissues][avrB][LaCl3_teatment] : A70.expr=abolished  Arath [systemic_tissues][avrB][DPD_treatment] : A70.expr=induced  Arath [EDS1,mut][avrB][local_tissues] : A70.expr=abolished  Arath [EDS1,mut][avrB][systemic_tissues] : A70.expr=abolished  Arath [SGT1B,mut][avrB][local_tissues] : A70.expr=none  Arath [SGT1B,mut][avrB][systemic_tissues] : A70.expr=abolished //A70.expr=attenuated  Arath [OPR3,mut][avrB][systemic_tissues] : systemic_immunity=attenuated  Arath [JIN1,mut][avrB][systemic_tissues] : systemic_immunity=attenuated  Arath [jasmonic_acid_treatment] : virulent_bacteria=restricted  Arath [avrB] : phloem_jasmonic_acid.accumulation=higher  Arath [hrpA] : phloem_jasmonic_acid.accumulation=lower  Arath [avrB] : phloem_salicylic_acid.accumulation=lower  Arath [hrpA] : phloem_salicylic_acid.accumulation=higher |
| 16132037 | Mechanisms of plant resistance to viruses. | Soosaar JL, Burch-Smith TM, Dinesh-Kumar SP. | Nat Rev Microbiol 2005 Oct;3(10):789-98 | R_genes = resistance_genes  R_proteins = resistance_proteins  NB = nucleotide_binding_site  LRRs = leucine_rich_repeats  NB_ARC = domains_of_resistance_genes //similar to the equivalent regions of the metazoan cell death genes Apaf-1 and CED4  NB_ARC_LRR = motives_required_for_nucleotide_binding_in_other_ATP/GTP_binding_proteins  R_genes (= NB_ARC_LRR_superfamily_of_plant_resistance_genes  Arath[RCY1] = resiatance_to_C_strain_Y_1  Arath[HRT] = HR_to_turnip_crinkle_virus  CMV = cucumber_mosaic_virus  TCV = turnip_crinkle_virus  Tobacco[N_gene] -> resistance_to_TMV  RCY1 -> resistance_to_CMV  HRT -> resistance_turnip_crinkle_virus  (resistance_protein, RNA_silencing) -|viral_pathogenesis  PCD = programmed_cell_death  programmed_cell_death (= hypersensitive_response  R_gene_mdiated_resistance  SAR  hypersensitive_response  RNA_silencing -> defence_agains_foreign_nucleic_acids  RCY1 |

**B) The controlled language: syntax specification** *(Excerpt from PhD thesis Steven Vercruysse, 2008)*

In this section we specify the variety of statements provided by MineMap. Most often, a *statement* is a representation of one single piece of information as captured from biological literature. For this description, it is impractical to fully report all the rules of possible combinations to form clauses and statements, in the way that the parser software is programmed. Instead, for user-friendliness, we will divide the general setup in topics, and for each topic give a few illustrative examples.

While most statements just represent one piece of information, like 'A -> B', for 'A stimulates B', two other constructs do not capture information. These are *mode definitions* or *comments*, which will be described before all other statements.

**1. Technical facilities**

Before using the language, it is good to know that one can insert a comment at any place. Comments are text that will be ignored by the parser program, and can be used to write down some reflections by the annotator. Note that this reflects how part of our inspiration also came from computer programming languages.

A -> B **//**… or: How I Learned To Stop Worrying and Love Writing the Thesis.

**/*** One is free to say what one wants

in a multi-line comment. ***/**

a_statement_that_spans_more -> **_**

than_one_line //Split single lines with a space+underscore.

**2. Mode definitions**

Mode definitions are statements that work on the meta-level; they attach their meta-information to all the statements that follow. For example, they can tell what species (organism) the current publication is describing, or what subject (e.g. section title in the article) is covered by the following statements.

**SPECIES:** Arabidopsis

**SETTING:** sucrose_starvation //The experimental setting.

**SUBJECT:** E2F role in G1/S transition //(This can be free text).

Note: the person who extracted the statements from a publication is also required meta-information, as well as a reference to the original publication. However, this shouldn't be defined as a statement; instead the MineMap web-interface will keep track of this information based on the user's login ID and the selected article's ID.

**3. Entities**

Entities are the words that build information-containing statements. In most computer languages and also in MineMap, a space is used to separate these entities. Therefore, if a term consists of multiple words, it should be separated by underscores ('_') instead of spaces. (In fact, this makes the parser program considerably easier).

**leaf_development**

**CDKA;1**

One can also combine separate bioentities via the 'dot-notation', to further specify an attribute of the first entity. For example, one can talk about the expression of the gene CycX: "CycX.expr". This dot should be read as the possessive form, so "CycX 's expression". Along the same line, this notation can also be used with a few language-specific shorthand attributes, like the "expr" for expression, "prot" for protein, "RNA", or "DNA".

CycX**.**expr

yeast**.**cdc2

time_interval**.begin**

A note concerning the gene vs. protein distinction: in several species (like Arabidopsis, but not human), a gene carries the same name as its derived protein. In that case, the bioentity name in MineMap will represent both at the same time, and usually the context will specify which one it is (e.g. only proteins get phosphorylated). Notice that in many cases this is not even clear from the publication, as even human annotators disagree in 23% of the cases (Tanabe 2005). If it is necessary to explicitly distinguish between the two, then one can use:

gene**.prot**

gene**.DNA**

Square brackets are used to define a derived entity from the basic one. The examples below represent: "phosphorylated Cdc25", "protein A phosphorylated at the site T14", and a double phosphorylated protein:

Cdc25**[**P**]**

A**[**P**,**T14**]**

A**[**P**,**T14**][**P**,**Y15**]**

One can attach a small, free-text note to an entity, between curly brackets:

Cdc25[P]**{**active form**}**

It should be noted that entities are always assumed to possibly be a set. For example when saying that CycD3 activates something, it means that every member of the *set of* CycD3s activates it. So when someone (later or earlier) defines that the entity CycD3 is actually a set, by saying that 'CycD3;1 is_a CycD3' and 'CycD3;2 is_a CycD3' etc., all these activates-relations would also hold for the members of that set.

One can also explicitly define a set, which is usually used together with the "="-operator:

**(**CycA**,** CycB**,** CycC**)**

One can declare all kinds of set combinations. With a little imagination, one easily sees that "u" stands for union, and "n" for intersection in the examples below. Note that our language should had to be both shorthand and typable on most keyboards. For the set-difference operator, we use the backslash symbol (the forward slash is used for mathematical division, see later on).

leaf **\** leaf_stoma

(A **u** B) **n** (C **\** (D, E) )

One can add a unit entity after a number, for example:

duration = 5 **h**

Some support for quantities is also present, like "# A", to be read as "number of A-s", and meaning the number of elements in the set A. It is in fact shorthand for a special attribute: "A.number_of". Also, some fuzzy quantities are predefined, like "high" and "low", which can for instance describe qualitative protein activity profiles coming from Western blots. Note that it is generally not possible to attach values to this fuzziness; they are only meant to describe relative changes.

**#** yeast.cyclins //(shorthand for the attribute 'number_of').

A = **high**

B = **medium**

C = **low**

**4. Relations**

The most basic relation, also used in many other information repositories (like ontologies), is the elementary parent-child relation, or "is_a" relation. For example, one can say that "CDKA;1 is a type of CDK". In MineMap this is written as "CDKA;1 (= CDK", with the mathematical set-inclusion as the relational symbol, reading out as "is a" or "subset of".

Note that both CDK and CDKA;1 should be thought of as representing *set*s here (with the latter as a singleton). Consider that it may be known that CDK comprises a number of different CDKA genes, CDKB genes, etc, and that there exists only one CDKA;1. But possibly in the future biologists could discover that there is again more than one type of CDKA;1. In any case, this is merely a conceptual matter.

CycD3;1 **(=** CycD3

(CycA, CycB, CycC) **(=** Cyclins

All the basic relational symbols are provided (equals, does not equal, larger than, etc) :

CycX.expr **=** high

A **!=** B //This is the common programming language operator "not equals".

A **>** B

A **<=** B

Homology between genes and proteins, or general similarity (a distinction can be made based on the context, the type of both entities) :

mouse.protA **=h** rabbit.protB

The most common activation relations are also available in the language. Note again that the "s" in the "-s>" operator is inspired by the step-up arrow as drawn in Kohn diagrams. As shorthand, the set notation can be used for each of the entities. For example "(A, B) ‑> C" would stand for "both A and B stimulate C". This statement is split into two separate pieces of information by the MineMap parser.

A **->** B //Activation stimulation (molecular interaction level unspecified).

(A, B) **->** C //Shorthand for: both A and B stimulate C.

A **-s>** B //Transcriptional activation (alike Kohn's notation).

A **-.>** B //Translational activation (alike Kitano's notation).

The inhibition relations are typed with almost the same symbols as the activations, except for the vertical bar symbol at the end '|' (also named *pipe*). Note that on most keyboards, the vertical bar symbol is depicted as a broken vertical bar '¦', to distinguish it from the 'I' (uppercase 'i') character. But when typed, it will likely appear as an un-broken '|'. The key is usually located next to the 'Enter' key, or on the '1' key.

A **-s|** B

The "~>" operator stands for "controls" or "mediates" and should be used when an influencing interaction is declared, but it was not defined whether this is an activation or inhibition. The perhaps less often used operator "-o" (alike Kohn's notation) stands for  "enzymatically promotes a transition", so in "A –o B", the B should not be a biomolecular process, for example a transformation.

A **~>** B //General

A **-o** (B -t> C) //(Notation like Kohn).

For transformations, one can use the "-t>" arrow. Although commonly drawn in interaction diagrams with the same plain arrow as for activation, we have to take away this ambiguity. For example in "A->B", A is the activator, while in "A -t> B", A is transformed.

A **-t>** B //Biochemical transformation from A to B.

A **-t>** A[P] //Phosphorylation of A.

A + B **-t>** C

A + B **-t>** C + D + E

We still mention some special shorthands:

Abc -> (P **->** A) //Abc stimulates the phosphorylation of A.

Abc -> (A **-t>** X) //Abc stimulates the destruction of molecule A.

Finally, "<->" declares the physical binding of two molecules, as used in Kohn maps. As mentioned before, by placing a letter in the middle ("<-z->") one can subsequently tell something more about the bound complex, all in the same statement.

A **<->** B

A **<-a->** B **,** a -| C //A and B bind, and the resulting complex inhibits C/

**5. Quantities**

It is also possible to perform some mathematics with entities:

(duration1 **+** duration2) **/** 2 > 5 h

cell_cycle.length **–** G1.length

cell_division_rate ***** duration

The "++" and "--" operators provide some convenient shorthand: "A++" is an identical alternative for "A = increased", and "B--" means "B = decreased". Note that "Increased" and "decreased" are both terms included in the PATO (phenotypic qualities) ontology.

cell_growth **++**

cell_division **– –**

**6. Time and space constraints**

The at-operator "@" is used to specify both temporal and spatial constraints. Whether it is space or time, can be deduced from the entity that follows the "@" symbol. On a historical note: in the original language specification, we provided both the "@T" and the "@L" operators (for time versus space). Not only provided this unnecessary overhead, we also noticed that the "@"‑notation was becoming useful to represent more than only time and space, but could be used for constraining-conditions in general. Therefore we dropped the T/L-appendix.

A <-> B **@** nucleus

A -s> B **@** S_phase //Postfix-notation.

**@** S_phase**:** A -s> B //Prefix-notation.

A -s> B **@** virus_infection //Non-space/time constraint.

As a special provision for the set combinations, we also allow terms for the universal spatial and temporal sets:

Abc.expr=high **@ allways** //= ... is true "at all times".

... @(**all**\Golgi) //= ... everywhere except in the Golgi app.

One can also use the space/time operator in the modifier part of an entity:

X**[@**t1**]** > X**[@**t2**]** //"X at time t1 is larger than at time t2".

Finally, when we take this manner of writing and we reuse the transformation symbol "‑t>", then we can define transportation, without inventing an extra operator. The following statement defines the transportation of Abc to the nucleus (literally, it would read out as: the transformation of Abc, to the Abc modified as being in the nucleus). Note that here, Abc's original location is not specified. However, this is often also not explicitly told in literature.

Abc -t> Abc**[@**nucleus**]**

**7. Prefixes**

One can specify, for a single statement, that it is valid only under certain special conditions. For example, to override the currently declared species (via a mode-definition)

yeast**:**  A -> sugar_intake //Overrides the currently declared species.

mouse**,** frog**,** chimp**:**  A -> B //Valid in all those species.

**species(**yeast**):**  A -> B //Alternate notation.

**setting(**drought_stress**):**  A -> B //Declares a special experimental setting.

Sometimes one may wish to enter an assertion that is only hypothesized in the publication. For this, one can use the specifier "HYP:" in front of the statement. While the information should be based on some leads, for now it is inconclusive.

**HYP:** A -> B @nucleus //The authors hypothesize that A activates B in the nucleus.

**8. Quantifiers and logic**

This section enters into the more experimental region of statements. First of all, we noticed that in literature, authors sometimes make general assertions like "This proves that there must be a protein that interacts with Abcd and that stimulates the G2/M-transition", or "Most of the CyclinZ-s interact with Abcd." To capture these, we included quantifiers (exists / for all) and logic in our language. For example the first statement would be written as:

**è** protein **:** **<->** Abcd **&** -> G2_M_transition

This, with the "è" operator (or "é") as the mathematical existence operator, looks a lot like a mathematical formula. As that is usually not too user-friendly, we made a first step in the user's direction, and allowed the omission of the 'quantified variable'. Concretely, here this means that one isn't obliged to write "è protein: protein <-> Abcd". So the slightly clearer "è protein: <-> Abcd" would read as "there exists a protein *that* binds to Abcd".

As a remark: although the current vocabulary accepts only accented é and è for the exists and the for-all quantifiers, this would best be replaced by a plain 'e' and 'a' in the future, because of keyboard generality and portability considerations.

Furthermore, like in mathematics, one can use logical operators in the tail of these statements. Possible logical operators are: & (and), | (or, the vertical pipe symbol again), ! (not, as in "!=" for inequality), "=>" (implies).

Some more examples:

**è** CDKB **:**  CDKB **<->** CycD4;1 **&** CDKB -> G2_M_transition

**è** CDKB **:** **<->** CycD4;1 **&** -> G2_M_transition

**à** CycD **:** **è** CDK **:** CycD **<->** CDK

An assertion like "Many cyclinD proteins bind to a CDKB protein" can not be captured easily in commonly known mathematical terms. It would be too weak to use the plain existence-operator "è", since we know that there exist *many* CycDs. Therefore we provided (experimental) "fuzzy quantifiers": one can take a quantifier-operator and append a modifier to it, like in:

**è[**many**]** CycD **:** <-> CDKB

Although to our knowledge, information like this can not be exported to any other format yet, still we believe it provides for an interesting idea.

**9. Various other statements**

We provide some basic support for defining transgenic phenotypes. The first example below says: "In an Arabidopsis CDKB1;1 overexpression line, cell division was decreased, but CycX's expression was high during the G1 phase". These statements don't provide direct molecular interaction evidence, but their indirectly implied clues can be useful for hypotheses about network structure, and for validating dynamical model simulations.

Arab**[**CDKB1;1**++]** **:** cell_division --, CycX.expr=high @G1

Mouse**[**A**++,** B**--]** **:** event, thing --, property ++, prop2 =, prop3 = equal

Further support for dynamical simulation comes in the form of experimentally measured time courses: gene expression profiles or protein activity profiles over time. These are virtually always given as fuzzy descriptions (often only visually), as is reflected in the capturing statement given below. Note that this already lies on the border line between *information* extracted from the paper, and raw, uninterpreted *data*.

Abc.expr **= [**G1.begin**:** low**,** G1**:** ++**,** S.begin **-**1h**:** medium**,** S.begin **+**1h**:** high**]**

Abc.expr **= [offset=**G1.begin**,** 0h**:** low**,** 0h-8h**:** ++**,** 8h-12h**:** =**,** 12-22h**:** --**]**

A last operator allows to negate any assertion, except for the special transgenics or time-course statements. The following says: "It is known that A does not activate B in any way".

**! (** A -> B **)**

**10. Review: Some basic reference examples**

**Mode-definitions**

SPECIES: Arabidopsis

SUBJECT: G1-entry

**Language basics**

(A,B,C) **(=** ABC //*Symbol* **(=** *means “subset of”, “is a”*

A **(=** B

CycD**.** expr = high //*Symbol* **.** *means “attribute”.*

A -t> A**[**P**]** //*Symbol* **[ ]** *means “modifier”.*

**Relations**

A = B *//A equals B.*

A =h B *//A is homologous to B.*

# CDKB > 2 *//The number of CDKBs is higher than 2.*

**@-specifications, sets**

A -> B *//A stimulates B.*

A -> B **@** G1 *//A stimulates B, during G1.*

cell_size++ **@** leaf \ leaf_stoma *//Cell size increases in the entire leaf except in the stomata.*

**Prefixes**

HYP: A -> B *//It is a hypothesis that …*

human: yeast.geneX -> geneY *//In human, the (inserted) yeast geneX stimulates (human) geneY.*

**Transformation**

B -t> C + D *//B is transformed (splitted) into C and D.*

A -t> A[P,T14] *//A becomes phosphorylated on site T14.*

**Quantifiers (exists / for all) & logic**

**è** CDKB **:** <-> CycD4;1 **&** -> G2_M_transition *//Some CDKB binds CycD4;1 & stimulates G2/M.*

**è[***many***]** CycD **:** <-> CDKB *//There are many CycDs that bind CDKB.*

**Transgenics & time courses**

Arab **[**CDKB1;1**++] :** cell_division --, CycX.expr=high @G1 *//In a CDKB1;1 overexpression line, …*

CycX.expr **= [**G1.begin**:** low**,** G1: ++**,** S.begin-1h**:** high**,** G2**:** --**]** *//A CycX expression time series.*
